# Supplementary material for: Repair of G1 induced DNA double-strand breaks in S-G2/M by alternative NHEJ
Source: Nat Commun. 2020 Oct 16;11:5239. doi: 10.1038/s41467-020-19060-w (PMC7567796; doi:10.1038/s41467-020-19060-w)
Supplement: Supplementary file 1 — Supplementary Information [file 41467_2020_19060_MOESM1_ESM.pdf]

Supplementary information

Repair of G1 induced DNA double-strand breaks in S-G2/M by alternative NHEJ

Wei Yu, Chloé Lescale, Loelia Babin, Marie Bedora-Faure, Hélène Lenden-Hasse, Ludivine Baron, Caroline Demangel, José Yelamos, Erika Brunet and Ludovic Deriano

Supplementary Figure 1

A

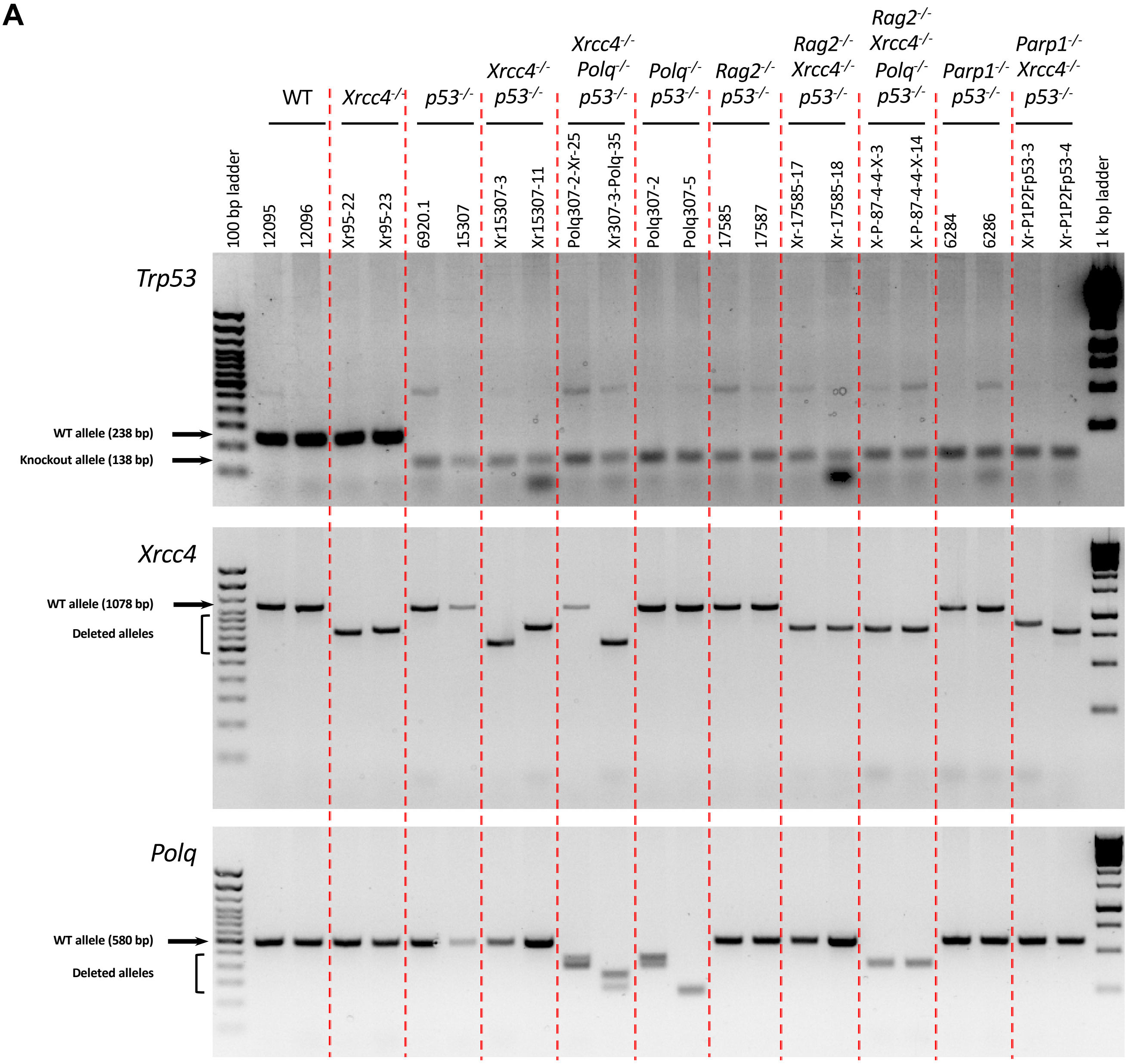

Supplementary Figure 1

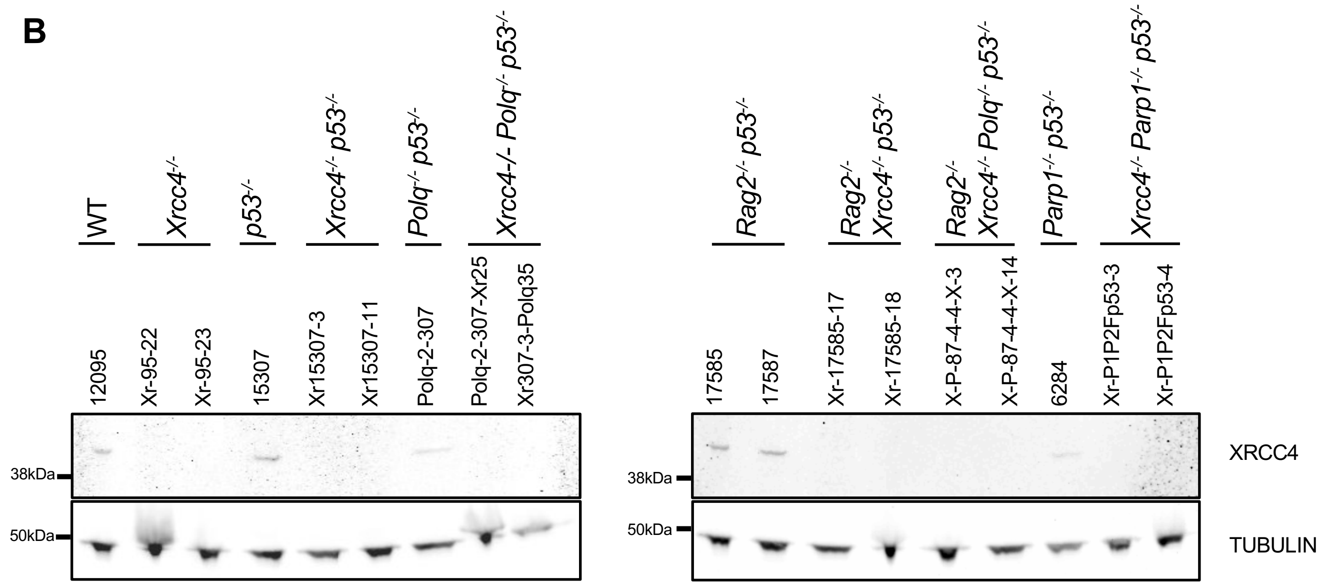

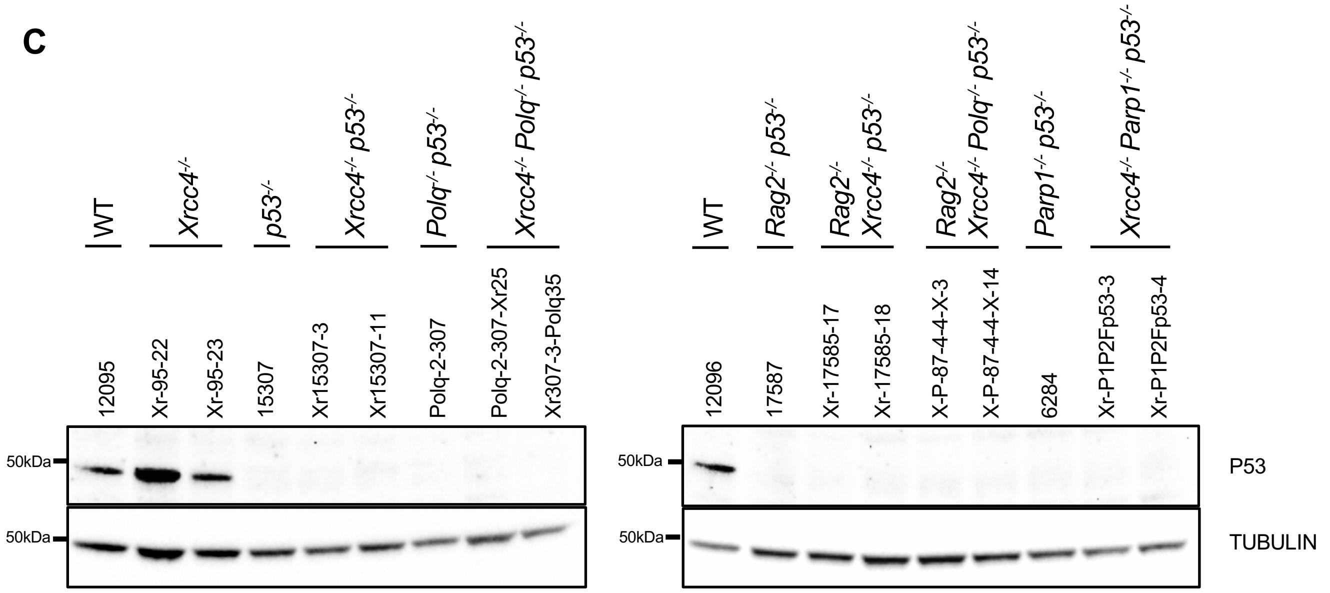

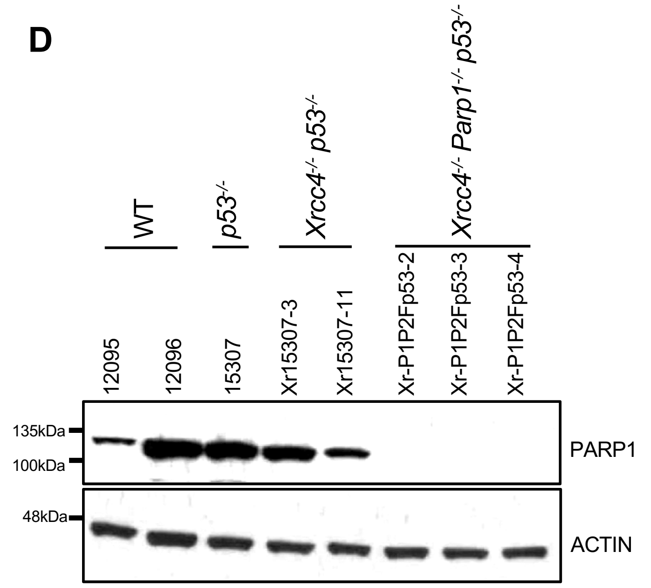

Supplementary Figure 1.
(A) PCR-based genotyping of indicated cell lines.
(B) XRCC4 Western blots for the indicated cell lines. γTubulin was used as a loading control.
(C) P53 Western blots for the indicated cell lines. γTubulin was used as a loading control.
(D) PARP1 Western blots for the indicated cell lines. ACTIN was used as a loading control. n=1 experiment.

Supplementary Figure 2

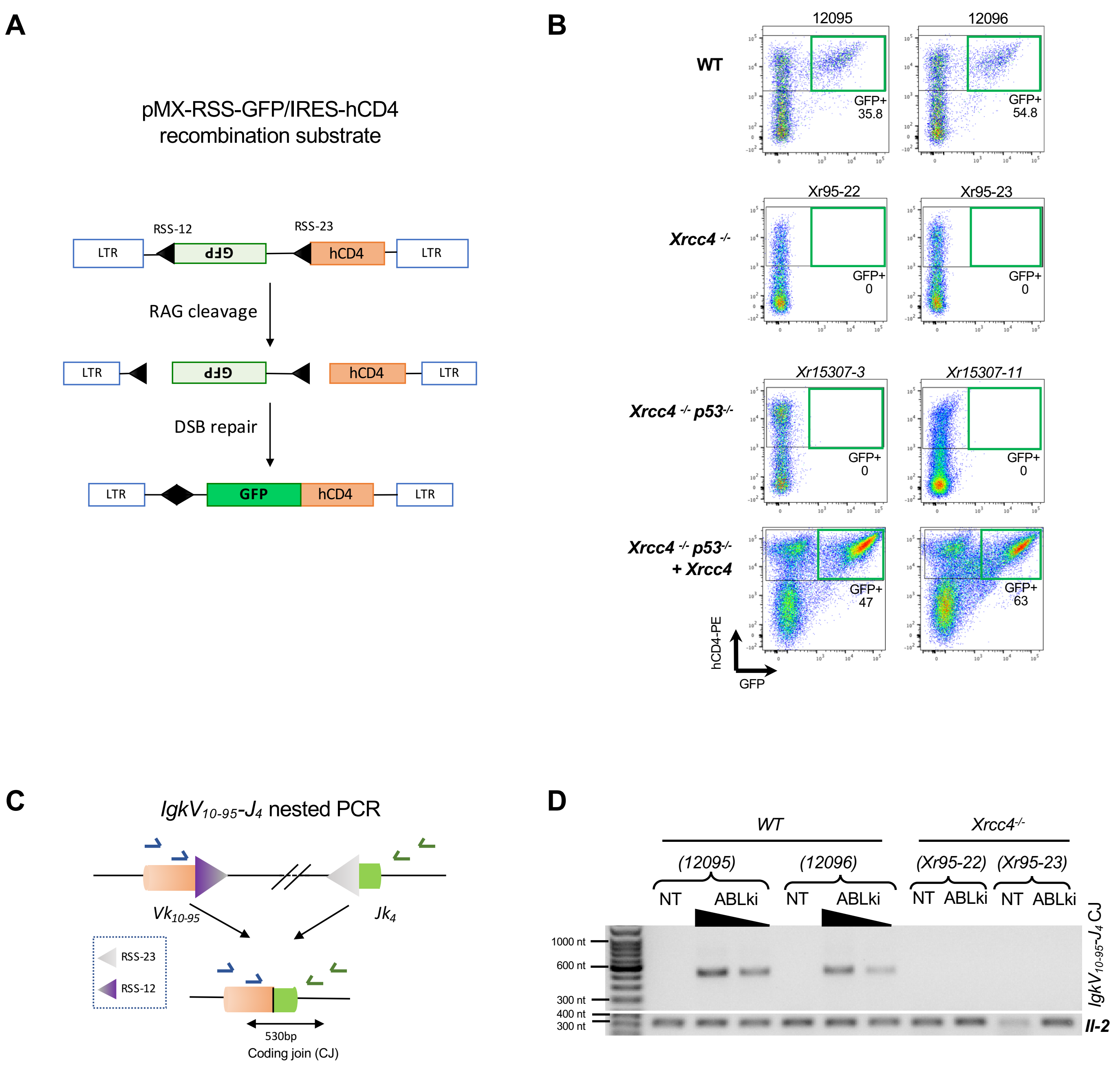

Supplementary Figure 2.
Related to Figure 1.
(A) Schematic representation of pMX-INV recombination substrate. The 12,23 -recombination signal sequences (black triangles), GFP cDNA, human CD4 cDNA (hCD4) and LTRs are indicated. **(B)** *v-Abl* pro–B cell lines treated for 72 hours with ABLki were assayed for PMX-INV rearrangement by flow cytometry. Representative plots are shown for 2 WT (*12095, 12096*), 2 *Xrcc4* $^{-/-}$ (*Xr95-22, Xr95-23*) and 2 *Xrcc4* $^{-/-}$ *p53* $^{-/-}$ (*Xr15307-3, Xr15307-11*) independent cell lines. The percentage of GFP^{+} cells among $hCD4^{+}$ cells is indicated. **(C)** Schematic representation of *IgkV*$_{10–95}$-*J*$_{4}$ coding join nested PCR strategy. **(D)** Semi-quantitative nested PCR analysis of *IgkV*$_{10–95}$-*J*$_{4}$ coding join from *v-Abl* pro-B cell lines in untreated conditions (NT) and after exposure to ABLki for 72 hours (ABLki). The PCR gel is representative of n=2 independent experiments.

Supplementary Figure 3

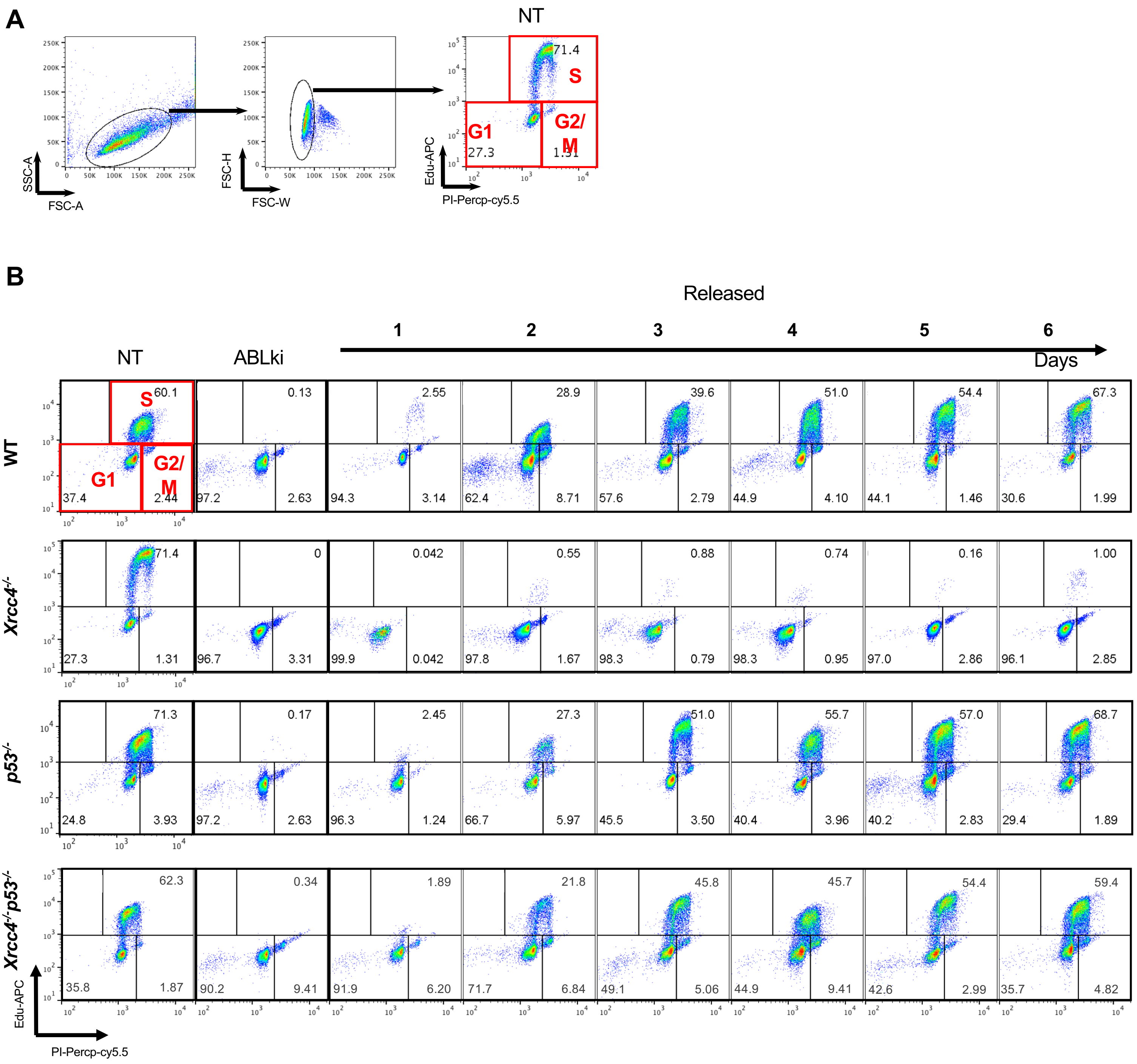

Supplementary Figure 3.
Related to Figure 1.
(A) Gating strategy to determine the percentage of cells in G1, S and G2/M phases.
(B) Cell cycle analysis of pro-B cells in untreated conditions (NT), after exposure to ABLki for 72 hours (ABLki) and one to six days after washing off ABLki (Released). For each condition, the percentages of G1 (lower left panel), S (upper right panel) and G2/M (lower right panel) cells among total live cells are indicated. Cell lines used: *WT (12095); Xrcc4$^{-/-}$ (Xr95-22); p53$^{-/-}$ (15307); Xrcc4$^{-/-}$ p53$^{-/-}$ (Xr15307-3).*

Supplementary Figure 4

A

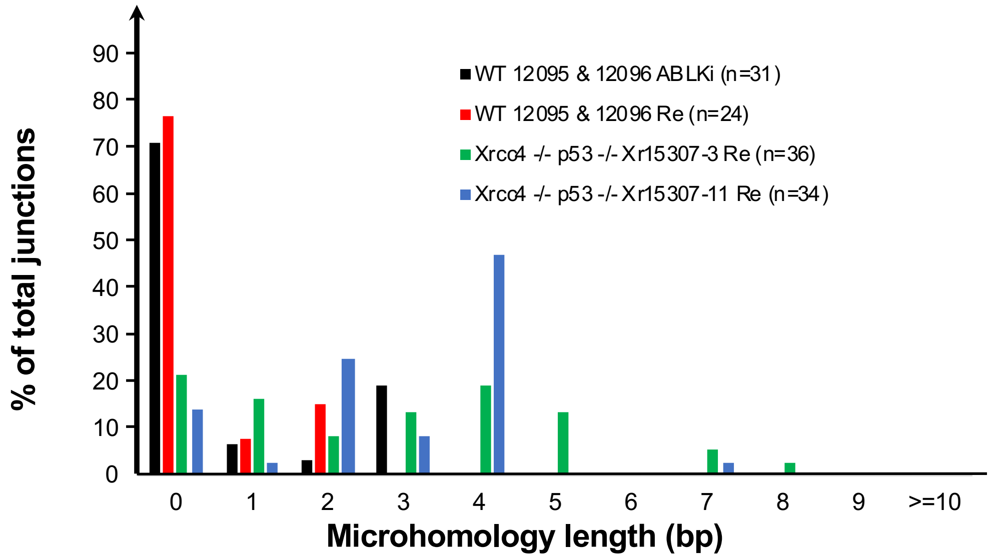

B

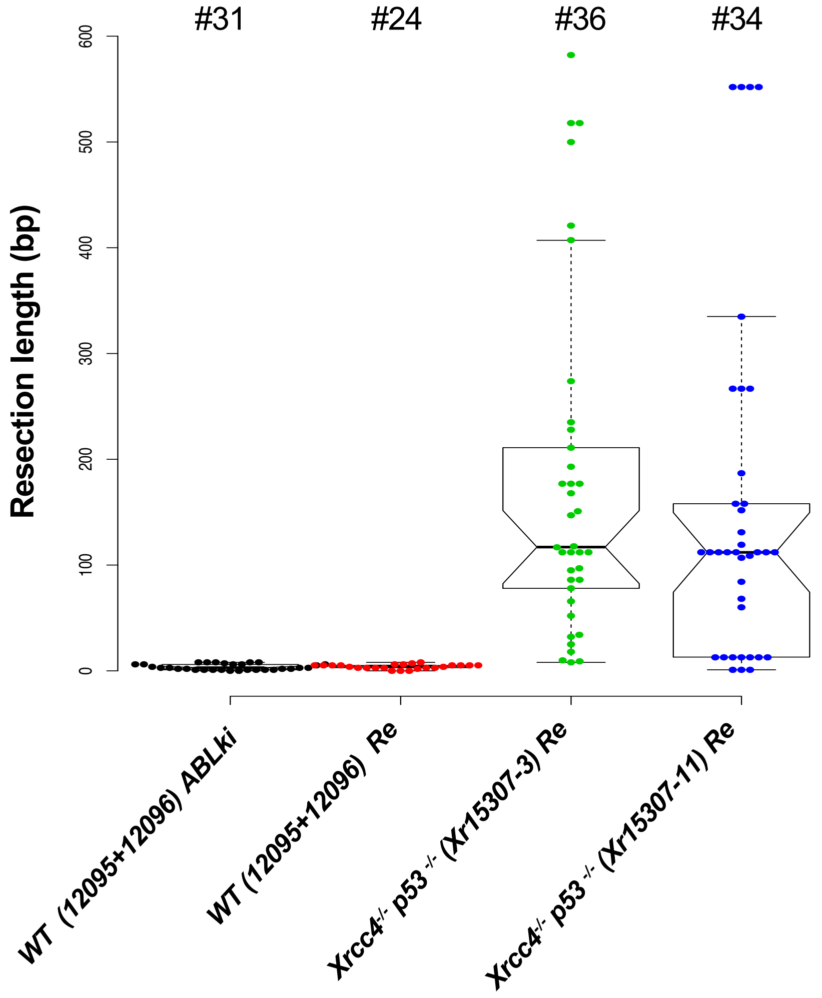

Supplementary Figure 4.
Related to Figure 1.
(A) Percentage of coding joins with variable length of microhomology. **(B)** Resection length of coding joins. Each dot represent one TOPO cloned sequence. The number of TOPO cloned sequences analysed is indicated above. Median values are in the center of box with bounds from 25 to 75 percentile, and the up and low whiskers are 1.5 times the range of the box bounds.

Supplementary Figure 5

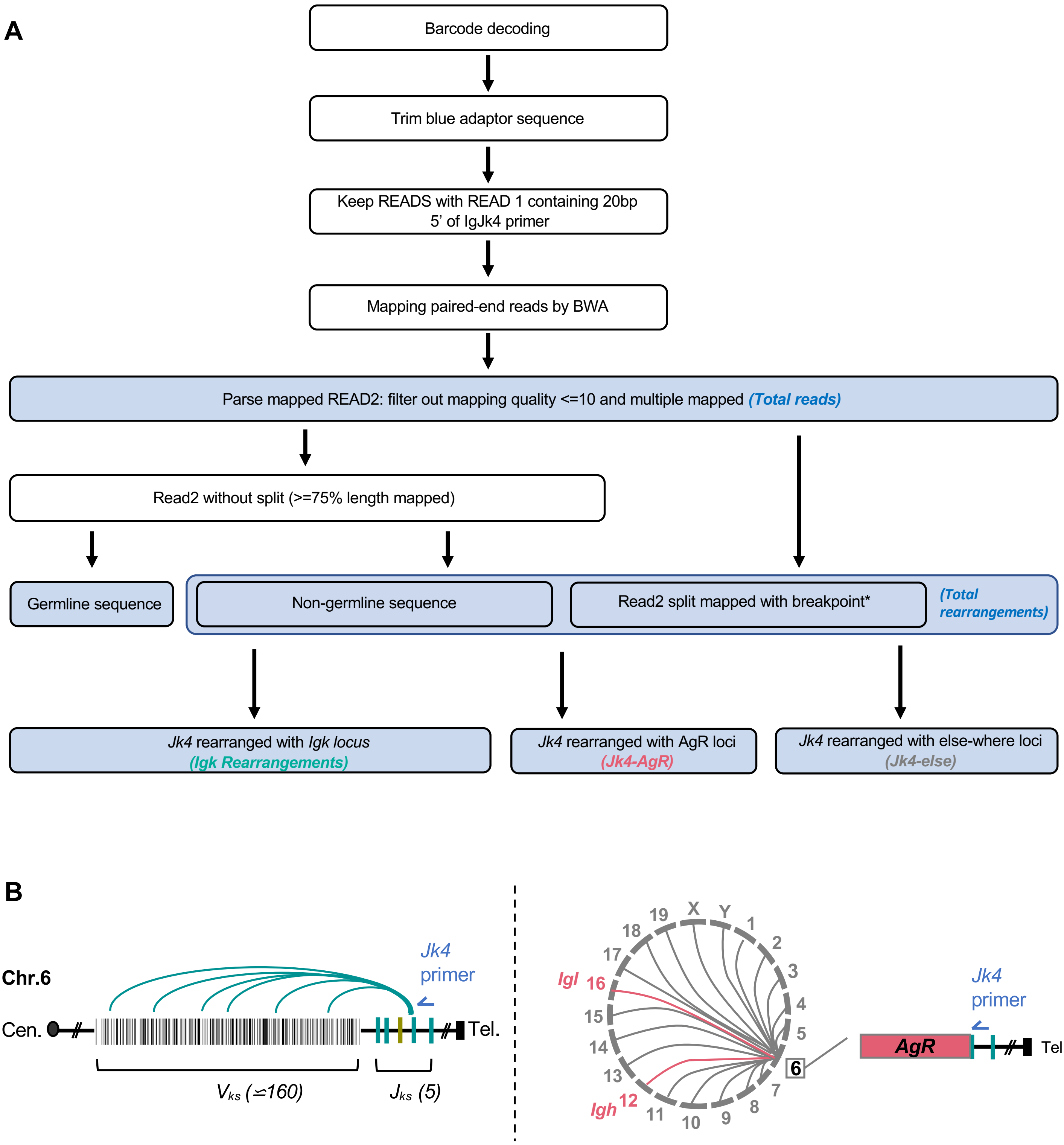

Supplementary Figure 5.
Related to Figures 1,2 and 3.
(A) LAM-HTGTS data analysis procedure. *Only reads containing read2 split mapped with breakpoint were used for the calculation of microhomology, insertion and resection. **(B)** Schematic representations of *IgkJ$_4$-V$_{region}$* junctions (left) and *IgkJ$_4$-AgR* translocations detected by LAM-HTGTS.

Supplementary Figure 6

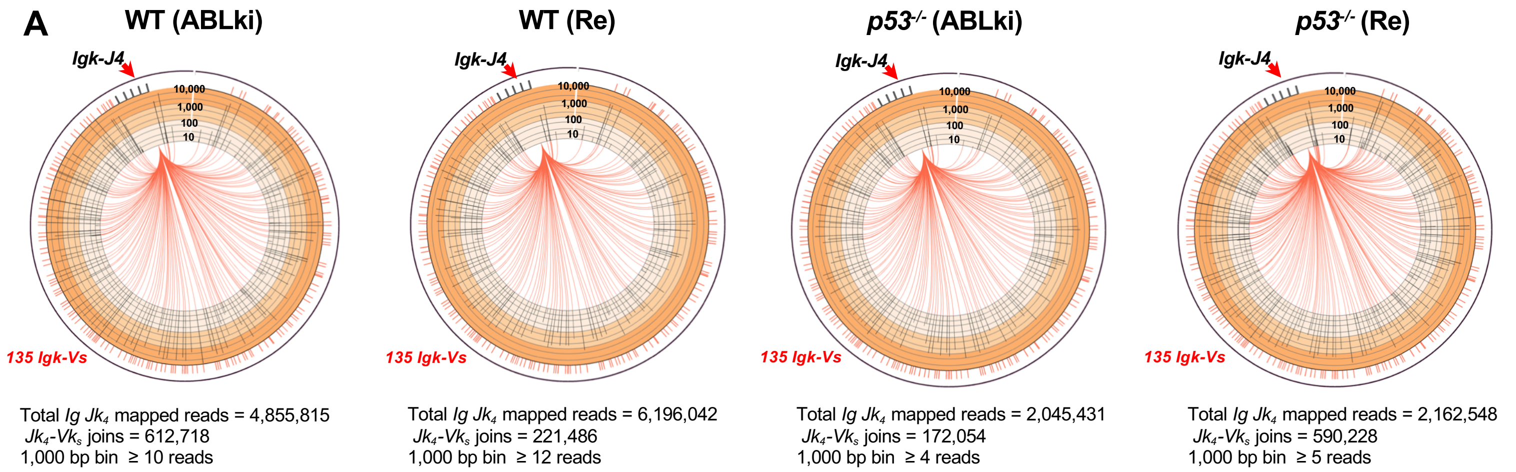

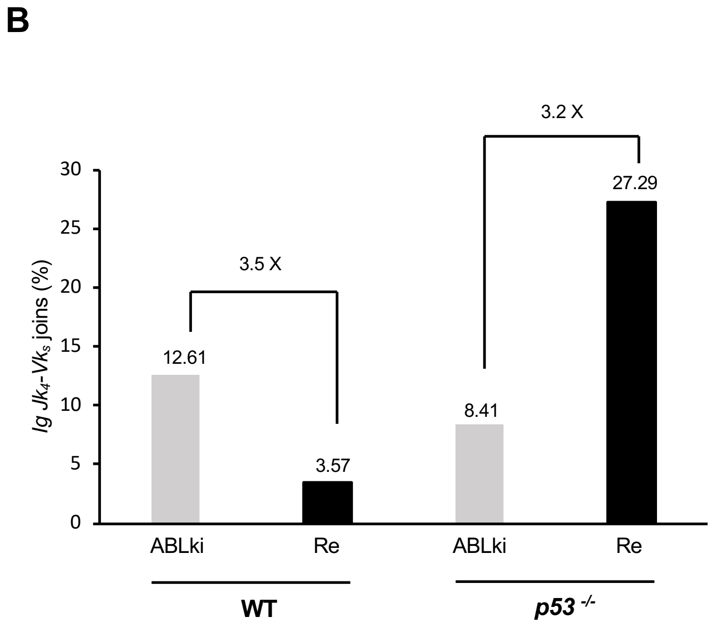

Supplementary Figure 6.
Related to Figure 1.
(A) Circos plots visualisation of *IgkJ*$_4$*-V*$_{region}$ junctions for G1 blocked (ABLki) and released (Re) *v-Abl* pro-B cells. *Igk* locus is divided into 1000 bps bins. Red lines indicate joins between bins and *Jk4* bait, with number of reads indicated below. The number of reads are plotted as black bar on a log scale with indicated custom ticks from 10 to 10000. Coordinates of *IgkV* and *J* segments are plotted as red and black bars at outmost zone. **(B)** Quantification of *IgkJ*$_4$*-V*$_{region}$ joins. Values are the percentages of translocations relative to total mapped reads. Fold enrichment in Released relative to ABLki is indicated above. Graph bars represent the pool of n = 4 independent experiments for WT G1 blocked cells, n=5 for WT released cells, n=3 for *p53*$^{-/-}$ G1 and released cells with two independent cell lines for each genotype.

Supplementary Figure 7

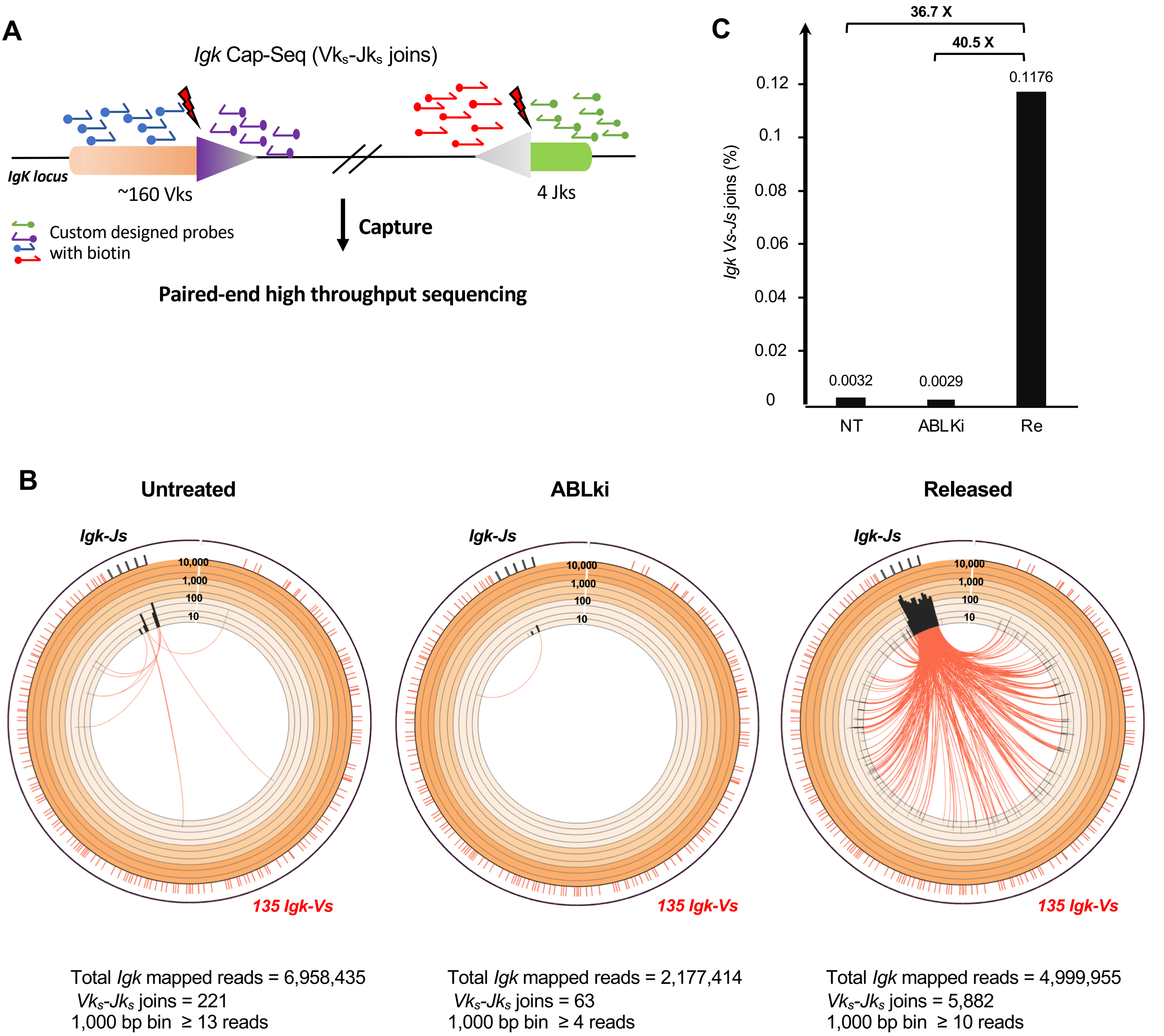

Supplementary Figure 7.
Related to Figure 1.
(A) Schematic representation of *IgkV*$_{region}$-*J*$_{region}$ Capture-Sequencing. **(B)** Visualization of *IgkV-J* joins within *Igk* loci by Circos plots for *Xrcc4* $^{-/-}$ *p53* $^{-/-}$ *v-Abl* pro-B cells. *Igk* locus is divided into 1000 bps bin linked by colored lines for bins with more than cutoff reads (NT: 13; ABLki: 4 ; Re: 10). Red lines: *IgkV*$_{region}$-*J*$_{region}$ joins. The number of joins are plotted as black bar on a log scale with indicated custom ticks from 5 to 1000. Coordinates of *IgkV* and *J* segments are plotted as red and black bars at outmost zone. **(C)** Quantification of *IgkV*$_{region}$-*J*$_{region}$ joins for *Xrcc4* $^{-/-}$ *p53* $^{-/-}$ *v-Abl* pro-B cells. Values represent the percentages of *IgkV*$_{region}$-*J*$_{region}$ relative to total mapped reads. Fold enrichment is indicated above. Graph bars represent n = 1 experiment for *Xrcc4* $^{-/-}$ *p53* $^{-/-}$ NT, ABLki and Re.

Supplementary Figure 8

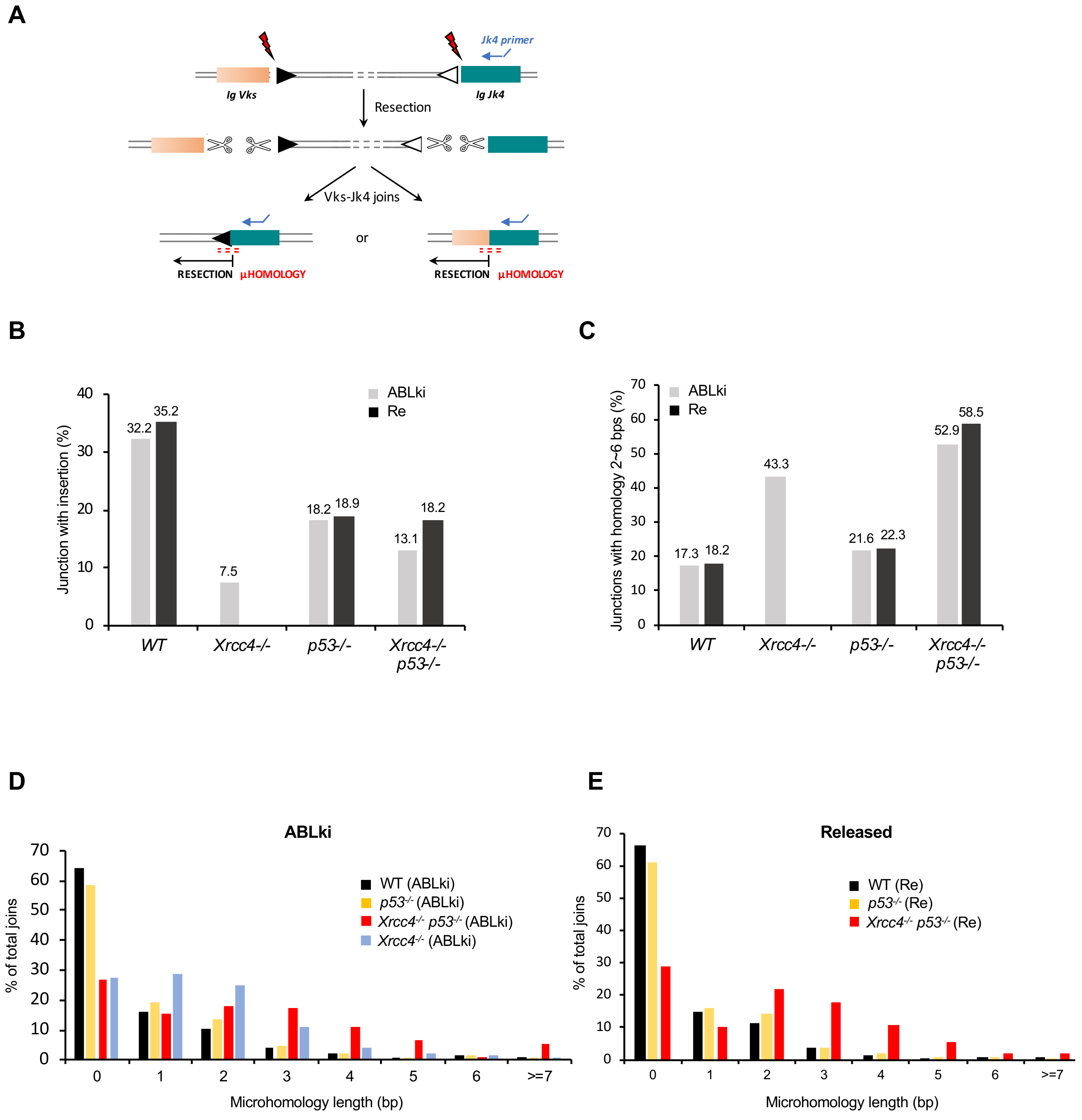

Supplementary Figure 8.
Related to Figure 2.
(A) Illustration of end resection and microhomology at *IgkJ*$_4$-*Vk*$_s$ junctions. **(B)** Percentage of *IgkJ*$_4$-*V*$_{region}$ joins with insertions. **(C)** Percentage of *IgkJ*$_4$-*V*$_{region}$ joins with 2-6 bps microhomology. **(D, E)** Microhomology length distribution of *IgkJ*$_4$-*V*$_{region}$ joins for ABLki treated (D) and released (E) *v-Abl* pro-B cells. Graph bars represent the pool of n = 4 independent experiments for WT G1 blocked cells, n=5 for WT released cells, n=3 for *p53*$^{-/-}$ G1 and released cells. n = 4 for *Xrcc4*$^{-/-}$ G1 blocked cells, n=3 for *Xrcc4*$^{-/-}$ *p53*$^{-/-}$ G1 blocked cells and n = 6 for *Xrcc4*$^{-/-}$ *p53*$^{-/-}$ released cells with two independent cell lines for each genotype.

Supplementary Figure 9

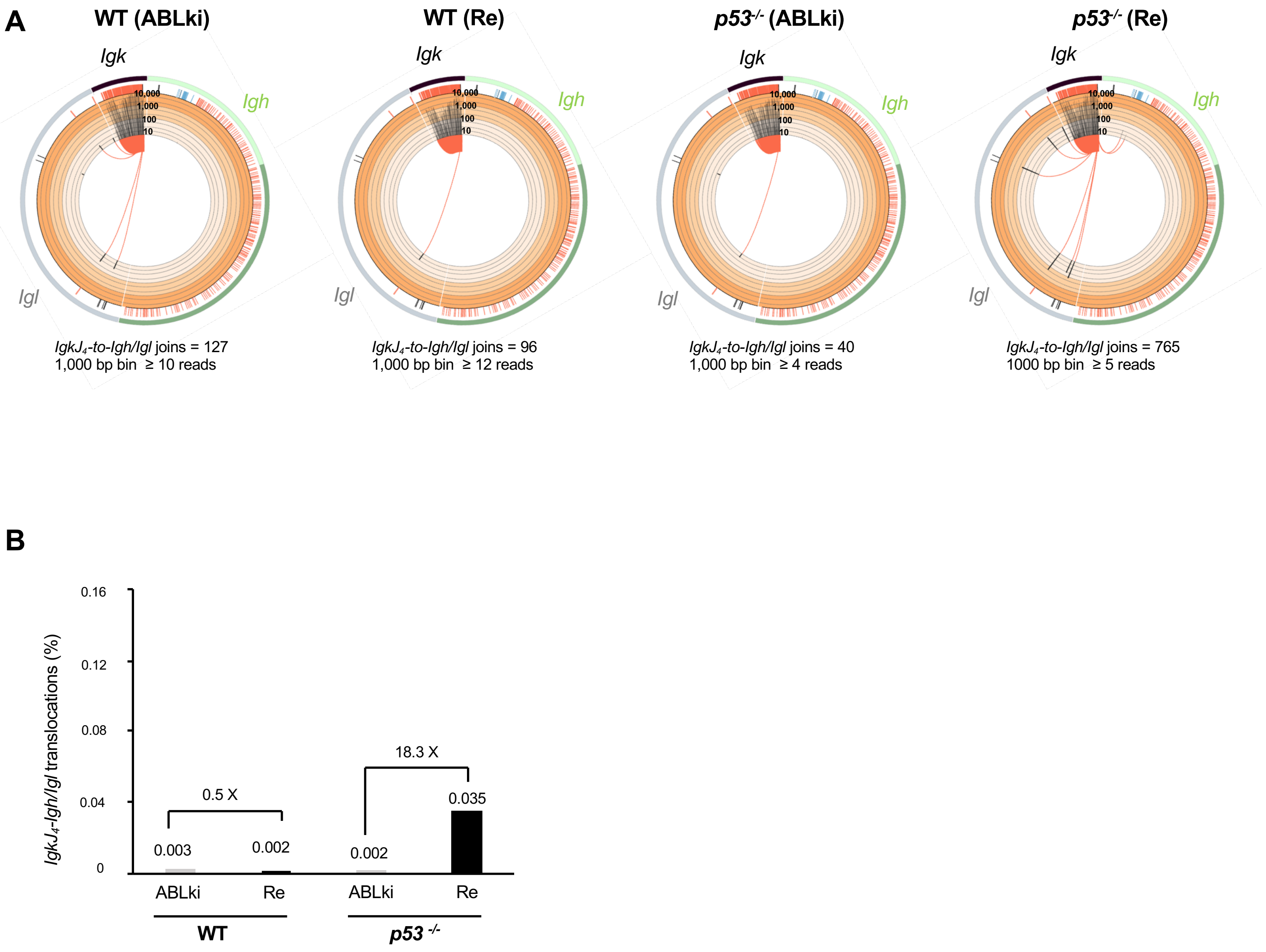

Supplementary Figure 9.
Related to Figure 3.
(A) Circos plots displaying *IgkJ*$_4$*-Igh/Igl* translocations. Translocations are represented as arcs originating from *IgkJ*$_4$ breaks with a minimum of indicated reads per 1,000bp bin for G1 blocked (ABLki) and released (Re) libraries. **(B)** Quantification of *IgkJ*$_4$*-Igh/Igl* translocations. Values are the percentages of translocations relative to total mapped reads. Fold enrichment in Released relative to ABLki is indicated above. Graph bars represent the pool of n = 4 independent experiments for WT G1 blocked cells, n=5 for WT released cells, n=3 for *p53*$^{-/-}$ G1 and released cells with two independent cell lines for each genotype.

Supplementary Figure 10

A

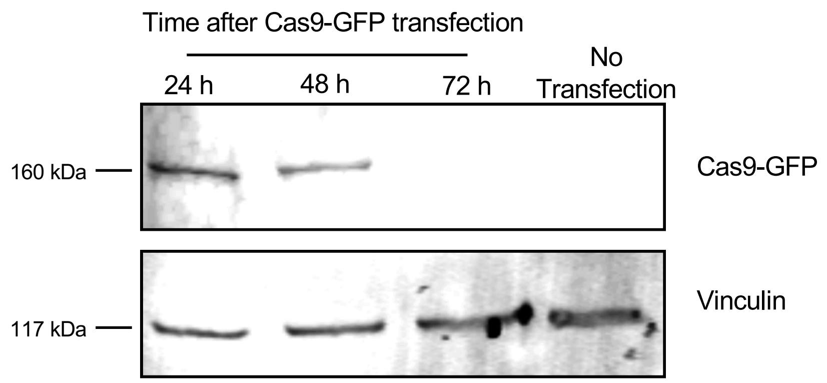

B

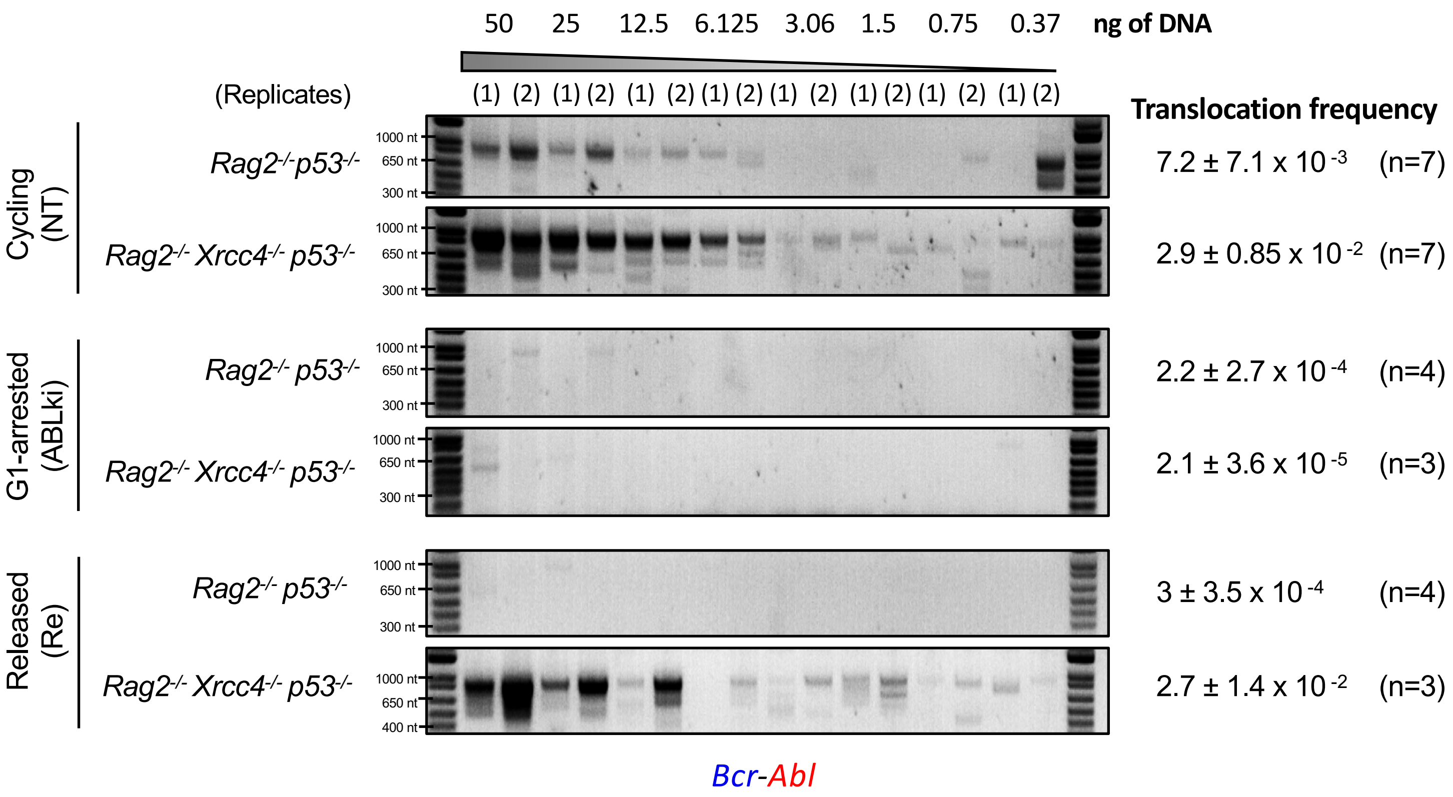

C

	Number of clones with unique breakpoint sequence	% MH	% Insertion	Resection length (Mean/Median)
Cycling Rag2-/- p53-/-	34	64.7	29.4	73.9/45
Cycling Rag2 -/- Xrcc4 -/- p53 -/-	68	61.7	30.8	79.9/29.5
Released Rag2-/- Xrcc4-/- p53-/-	56	69.6	23.2	107.5/46

Supplementary Figure 10

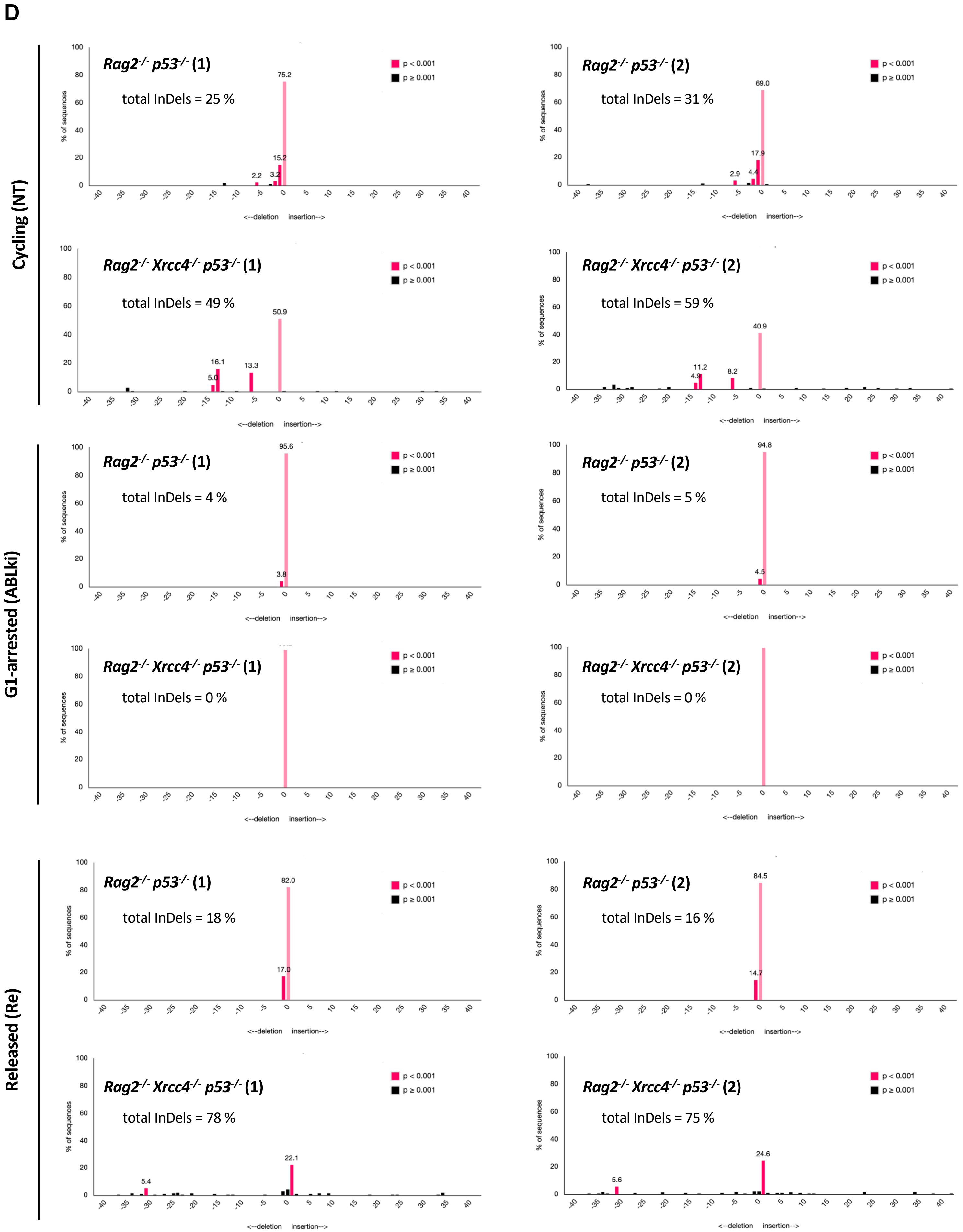

Supplementary Figure 10

E

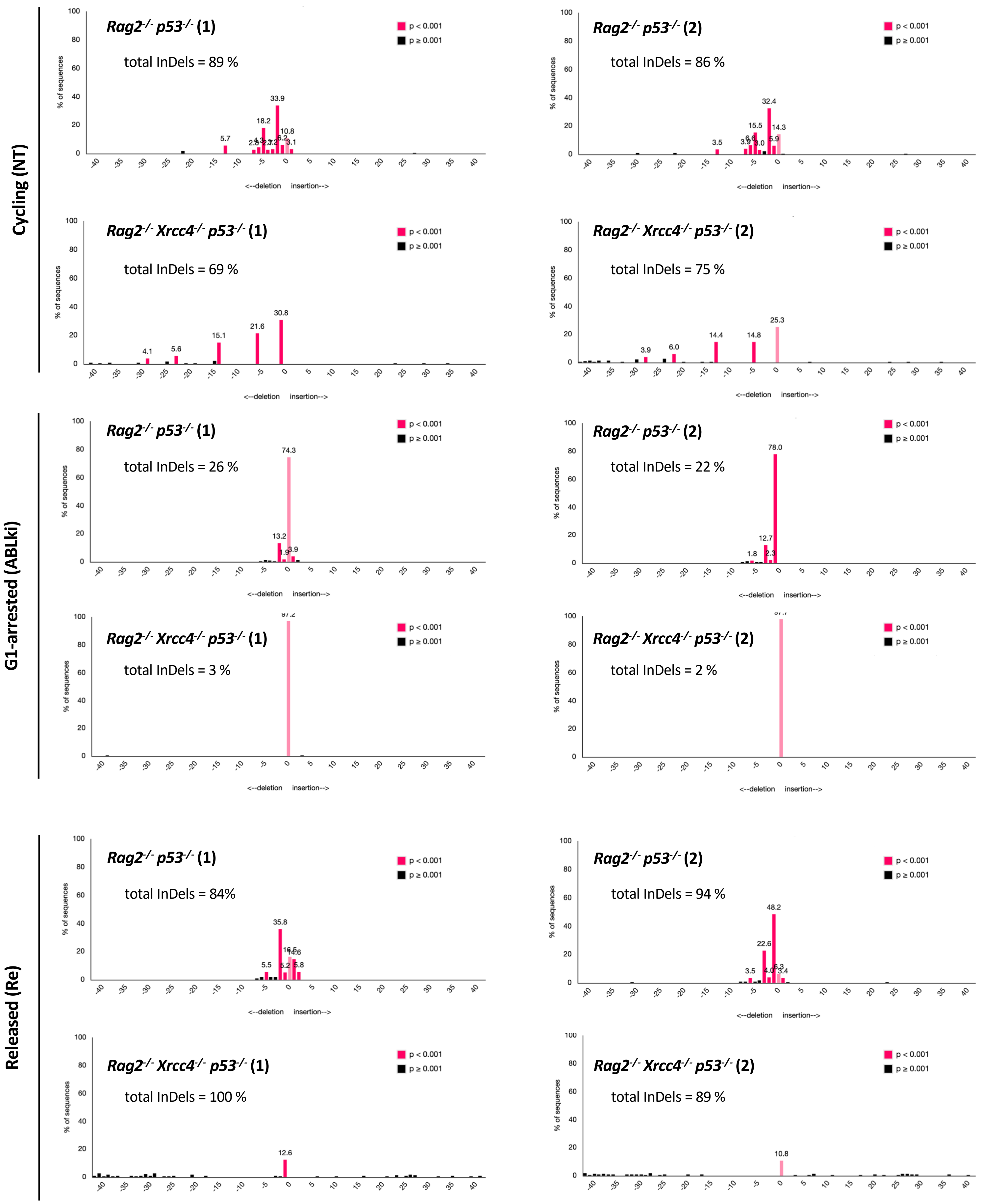

Supplementary Figure 10

Supplementary Figure 10.
Related to Figure 3.
(A) Western blot showing Cas-GFP expression at 24 hours (h), 48h and 72h after transfection of ABLki-treated pro-B cells. Vinculin was used as loading control. The blot is representative of n=2 independent experiments.
(B) Nested PCR for *Bcr-Abl* translocation detection for each genotype on DNA serial dilutions (2 biological replicates for each dilution indicated as (1) and (2)). Cell lines used: *Rag2*$^{-/-}$ *p53*$^{-/-}$ (17585, 17587); *Rag2*$^{-/-}$ *Xrcc4*$^{-/-}$ *p53*$^{-/-}$ (Xr-17585-17, Xr-17585-18). Number of cells corresponding to each dilution is listed at the top of the gel (a diploid cell contains ~6 pg of DNA). Total amount of cells amplified for each genotype: 2x(50+25+12.5+6.125+3.06+1.5+0.75+0.37)/6x10^3=33101 cells. Calculation of translocation frequency : f>= number of PCR amplicons / number of amplified cells as described in Renouf et al, Methods Enzymol., 2014. Indicated translocation frequencies are the mean ± standard deviation of all transfections for each genotype with the number (n) of experiments indicated.
(C) Topo cloning and sequencing analysis of *Bcr-Abl* PCR products with resection length, microhomologies (MH) and insertions at *Bcr-Abl* translocation breakpoint sites (see also Supplementary Table 5). **(D-E)** Graphical representation of indels frequency for each genotype generated with TIDE: Tracking of Indels by Decomposition (https://tide.deskgen.com/). n=2 independent experiments with two independent cell lines for each genotype **D.** *Abl* breakpoint junctions. **E.** *Bcr* breakpoint junctions. P-values were calculated using a Two-sided Pearson's chi-squared test.

Supplementary Figure 11

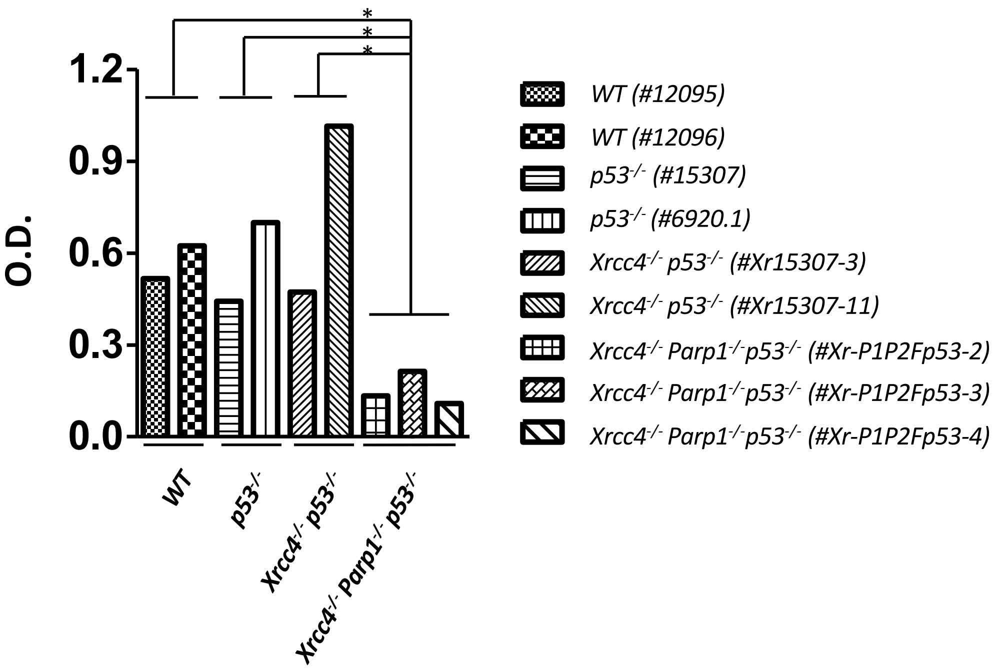

Supplementary Figure 11.
Related to Figure 4.
PARP activity in *v-Abl* pro-B cell lines. * $p < 0.05$ (Two-sided Student's t-test). WT vs *Xrcc4$^{-/-}$ Parp1$^{-/-}$ p53$^{-/-}$* : P=0.0013; *p53$^{-/-}$* vs *Xrcc4$^{-/-}$ Parp1$^{-/-}$ p53$^{-/-}$* : P=0.0094; *Xrcc4$^{-/-}$ p53$^{-/-}$* vs *Xrcc4$^{-/-}$ Parp1$^{-/-}$ p53$^{-/-}$*: P=0.037.

Supplementary Figure 12

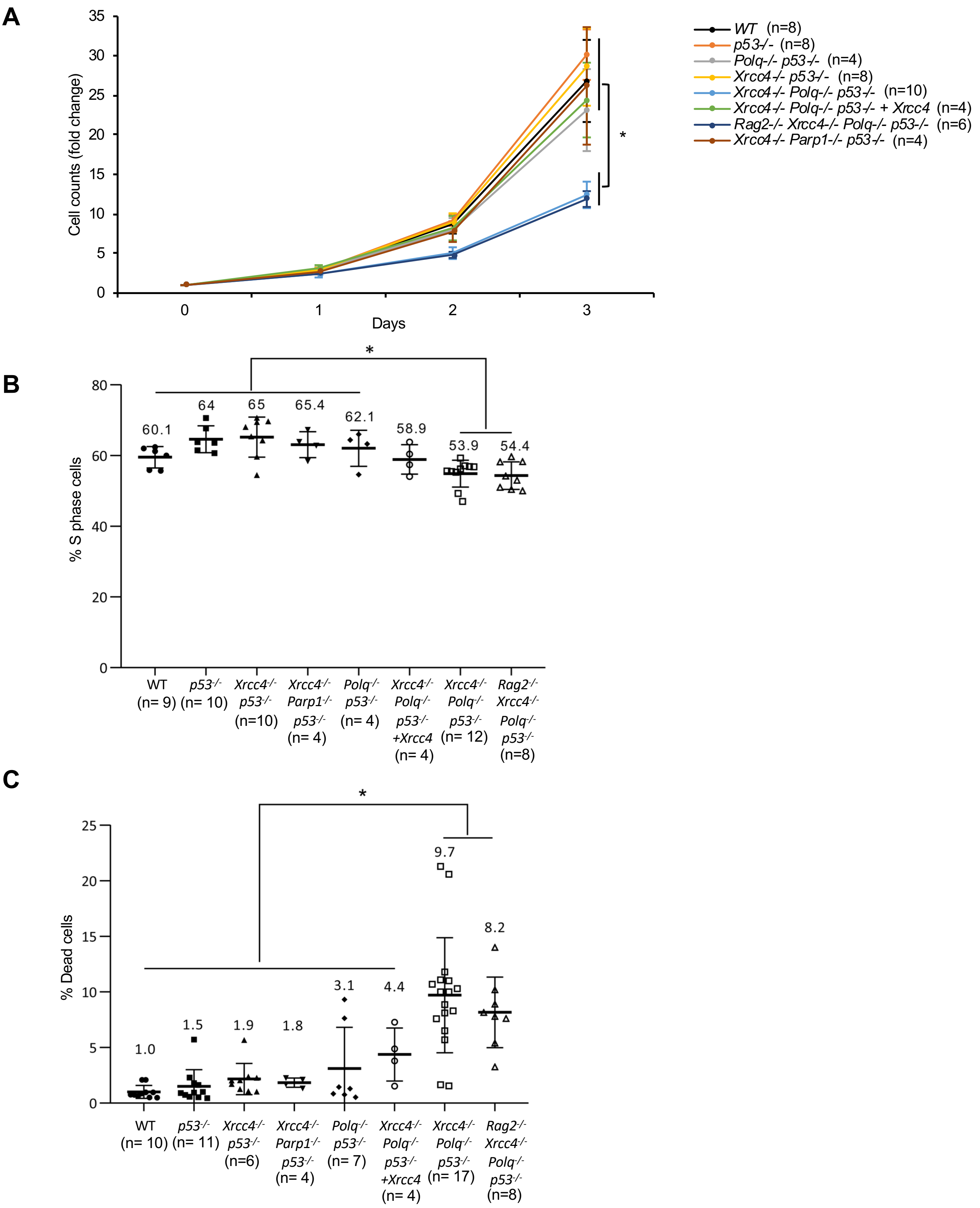

Supplementary Figure 12.
Related to Figure 4.
(A) Untreated cells were plated at 0.5 x 10^6 cells/ml and then counted every 24hr. * $p < 0.05$ (Two-sided Student's t-test) at day 3.). The number of independent experiments for each genotype is indicated into brackets. **(B)** Percentage of S phase cells (measured by Edu-PI cell cycle staining) in untreated cells. * $p < 0.05$ (Two-sided Student's t-test).).The number of independent experiments for each genotype is indicated into brackets. **(C)** Percentages of dead cells in untreated cells. All data represent the mean ± standard deviation. * $p < 0.05$ (Two-sided Student's t-test). The number of independent experiments for each genotype is indicated into brackets. Two or three isogenetic clones were analysed for each genotype. Source data are provided as a Source Data file.

Supplementary Figure 13

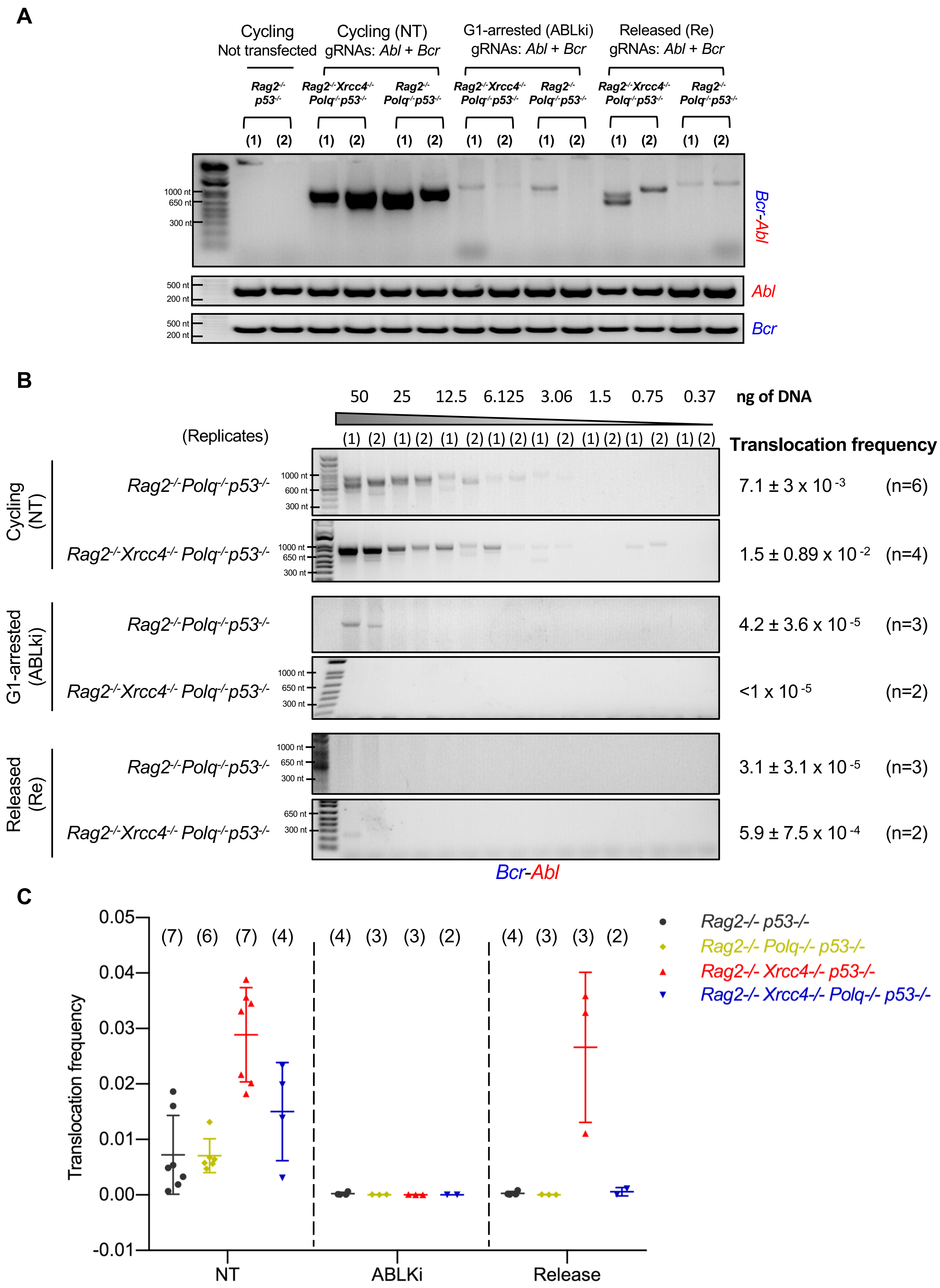

Supplementary Figure 13

D

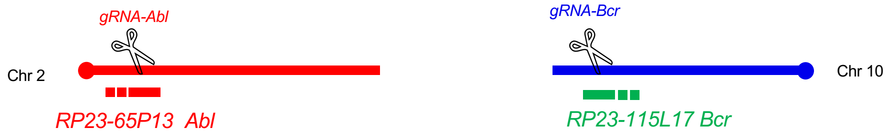

E

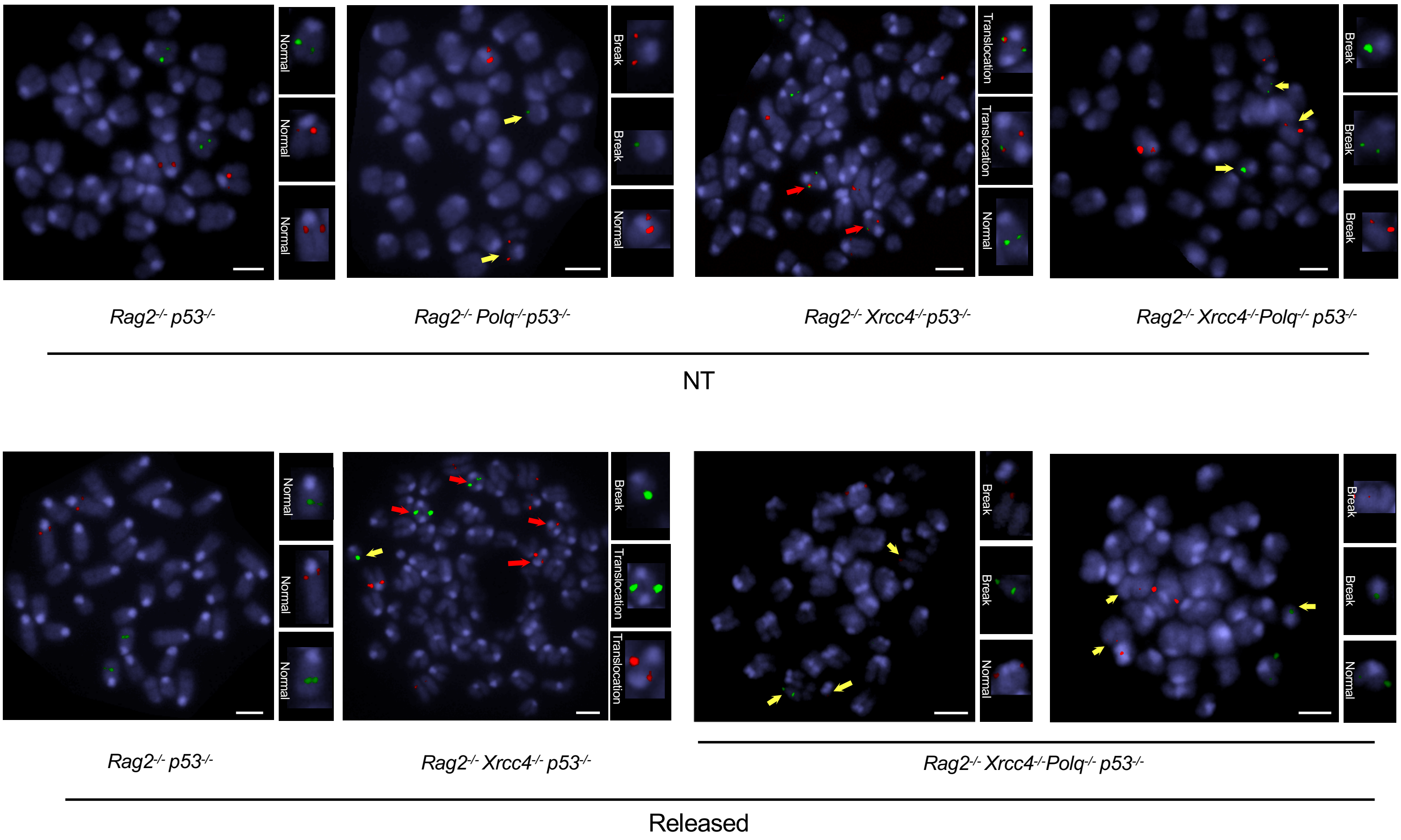

F

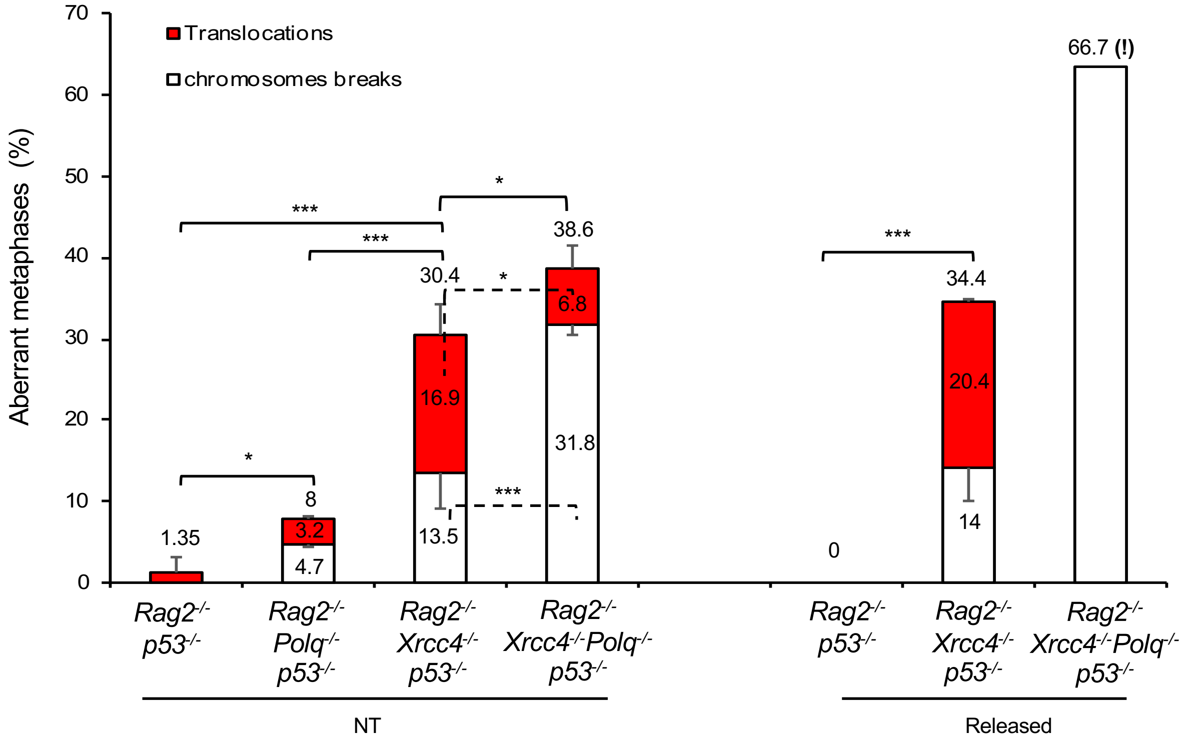

Supplementary Figure 13.

(A) Representative PCR amplifications of *Bcr-Abl* translocation breakpoints; in untreated cycling (NT) (transfected or not with Cas9 and gRNAs), G1-arrested (ABLki) and released/cycling (Re) *v-Abl* pro-B cells of the indicated genotype. PCR of *Abl* and *Bcr* loci were used as controls. Cell lines used: *Rag2$^{-/-}$ p53$^{-/-}$ (17585,17585); Rag2$^{-/-}$ Xrcc4$^{-/-}$ Polq$^{-/-}$ p53$^{-/-}$ (X-P-87-4-4-X-3, X-P-87-4-4-X-14); Rag2$^{-/-}$ Polq$^{-/-}$ p53$^{-/-}$ (Polq-17587-8, Polq-17587-13).* The number of independent experiments is indicated in Supplementary Figure 13C.

(B) Direct PCR for *Bcr-Abl* translocation detection for each genotype on DNA serial dilutions (Two biological replicates for each dilution indicated as (1) and (2)). Number of cells corresponding to each dilution is listed at the top of the gel (a diploid cell contains ~6 pg of DNA). Total amount of cells amplified for each genotype: $2x(50+25+12.5+6.125+3.06+1.5+0.75+0.37)/6x10^3=33101$ cells. Indicated translocation frequencies are the mean ± standard deviation of all transfections for each genotype and the numbers of transfection are indicated in the right of panel.

(C) Dot plot of translocation frequencies after GFP-Cas9 transfections in untreated cycling (NT), G1-arrested (ABLki) and released/cycling (Re) *v-Abl* pro-B cells *.* Each dot indicates one transfection. Bars represent means ± s.d. from at least two independent experiments performed with a minimum of two independent B cell clones. The number of independent experiments is indicated into brackets.

(D) Schematic representation of the *Bcr* and *Abl* loci, with positions of the BACs used for generation of DNA FISH probes indicated.

(E) Representative metaphases from cycling (NT) and released *Rag2$^{-/-}$ p53$^{-/-}$* , *Rag2$^{-/-}$ Polq$^{-/-}$ p53$^{-/-}$, Rag2$^{-/-}$ Xrcc4$^{-/-}$ p53$^{-/-}$* and *Rag2$^{-/-}$ Xrcc4$^{-/-}$ Polq$^{-/-}$ p53$^{-/-}$ v-Abl* pro-B cells with *Abl* BAC probe (red), *Bcr* BAC probe (green). Yellow arrowheads point to broken and orange arrowheads for translocation. Scale bars = 3μm. The list of independent experiments is provided in Supplementary Table 6.

(F) Percentage of aberrant metaphases from cycling (NT) and release *v-Abl* pro-B cells of the indicated genotypes. Cycling cells were harvested 4 day after transfection. Released cells were harvested 5 days after washing off ABLki. Histograms represent means ± s.d. from at least two independent experiments performed with a minimum of two independent B cell clones. The mean percentage of total aberrant metaphases is indicated above. The mean percentages of metaphases with translocations (red) and chromosomes breaks (white) are indicated (See also Supplementary Table 6). The black lines are the statistic of aberrant cells vs normal cells. The dash lines are the statistic of cells with break vs other cells (normal metaphases + metaphases with translocations) and cells with translocation vs other cells (normal metaphases + metaphases with breaks). **P*<0.05; ****P*<0.001 (Two-sided Fisher exact test) (See also Supplementary Table 6). (**!**) A total of 12 metaphases were obtained for this condition (0 metaphase with *Bcr-Abl* chromosomal translocations and 8 metaphases with *Bcr* and/or *Abl* chromosome breaks) and thus statistical analysis was not performed (The list of independent experiments for each conditions is provided in Supplementary Table 6).

Supplementary Figure 14

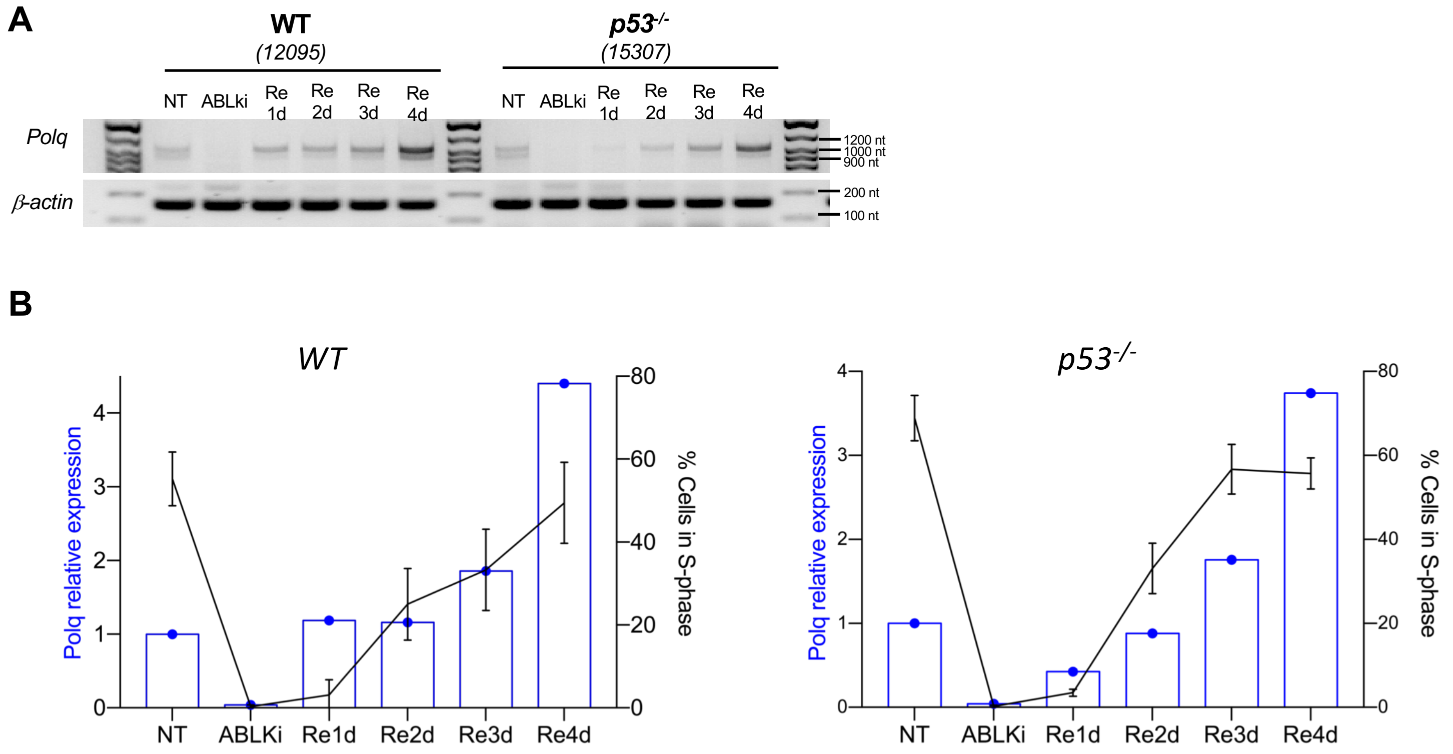

Supplementary Figure 14.
Related to Figure 5.
(A) *Polq* mRNA expression in untreated (NT), G1-blocked (ABLki) and released (Re1d to Re4d) WT *and p53*$^{-/-}$ *v-Abl* pro-B cells. *β-actin* mRNA was used as a control. Cell lines used: WT(*12095*); p53$^{-/-}$ (*15307*). **(B)** Quantification of *Polq* mRNA expression (blue bar graphs, left y axis) and the percentage of S-phase cells (black curve graphs, right y axis, lines represent means ± S.D. from a minimum of two independent B cell clones) in WT and *p53*$^{-/-}$ *v-Abl* pro-B cells. NT : untreated; ABLki: treated for 72 hours with ABLki; Re1d to Re4d: released. The bar plots of Polq expression represent the quantification of n=1 independent experiment from panel A.

Supplementary Figure 15

A

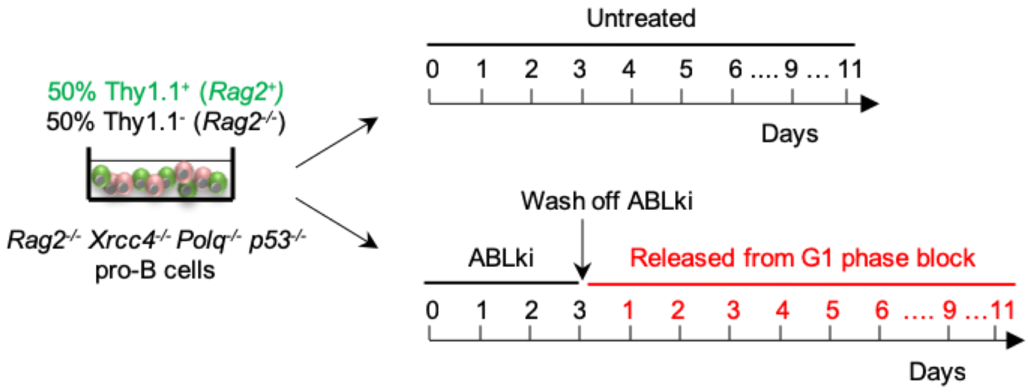

B

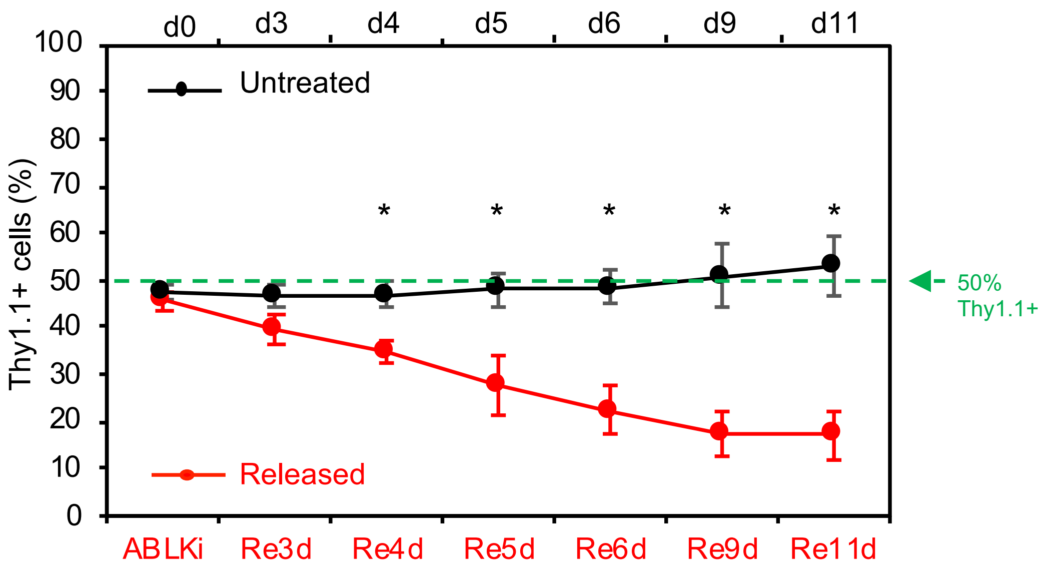

Supplementary Figure 15.
Related to Figure 5.
(A) RAG2 complemented Thy1.1^{+} and un-complemented Thy1.1^{-} *Xrcc4* $^{-/-}$ *Polq* $^{-/-}$ *p53* $^{-/-}$ *v-Abl* pro-B cells were mixed at a ratio of 1:1 and the percentage of Thy1.1^{+} cells during normal cell culture conditions (Untreated) and after release from ABLki treatment (Released) was analyzed by FACS at different time points. **(B)** Quantification of live Thy1.1^{+} (Rag2+) and Thy1.1^{-} (*Rag2*$^{-/-}$) *Xrcc4* $^{-/-}$ *Polq* $^{-/-}$ *p53* $^{-/-}$ *v-Abl* pro-B cells at different time points during normal cell culture conditions (Untreated) and after release from ABLki treatment (Released). Dots represent mean ± SEM from two experiments performed with two independent cell lines. * p<0.05 (Two-sided Student's t-test). Cell lines used: *Rag2*$^{-/-}$ *Xrcc4*$^{-/-}$ *Polq*$^{-/-}$ *p53*$^{-/-}$ *(X-P-87-4-4-X-3, X-P-87-4-4-X-14).* Re4d vs d4: *P*=0.0043; Re5d vs d5: *P*=0.0096; Re6d vs d6: *P*=0.0018; Re9d vs d9: *P*=0.00067; Re11d vs d11: *P*=0.00093.

Supplementary Figure 16

A

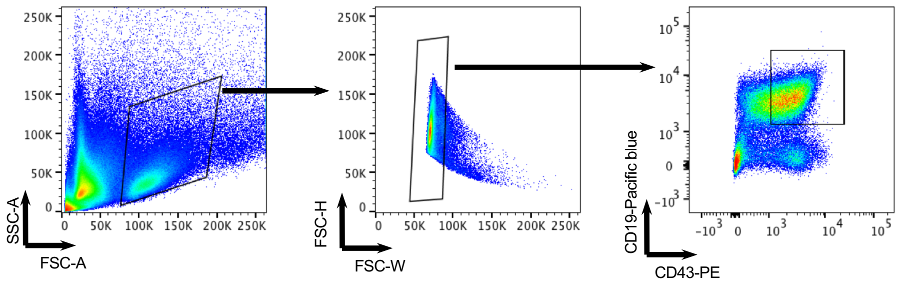

B

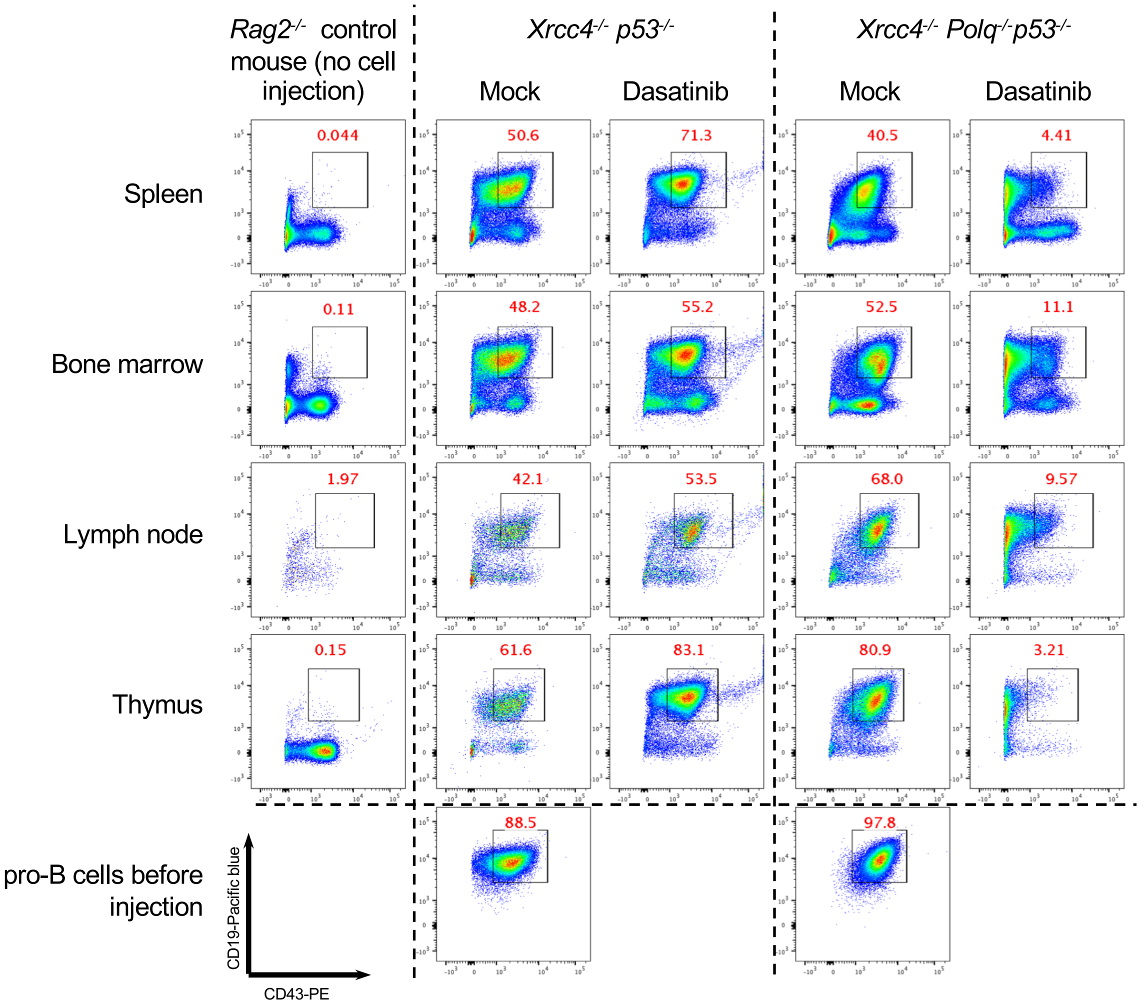

Supplementary Figure 16.
Related to Figure 5.
(A) Gating strategy to determine the percentage of $CD19^+$ $CD43^+$ leukemic cells.
(B) Representative plots of lymphoid organs obtained from sick mice injected with *v-Abl* pro-B leukemic cells. Numbers in red indicate the percentage of $CD19^+$ $CD43^+$ leukemic cells among live single cells.

Supplementary Table 1

List of cell lines used in the study

Genotype	Clone ID	Allele 1	Allele 2	Generation Method	Origin	Reference
WT	12095	NA	NA	v-Abl/Bcl2 immortalization of bone marrow pro-B cells	Mouse	Lescale C. et al., Nat Com. 2016
	12096	NA	NA	v-Abl/Bcl2 immortalization of bone marrow pro-B cells	Mouse	Lescale C. et al., Nat Com. 2016
p53 -/-	6920.1	NA	NA	v-Abl/Bcl2 immortalization of bone marrow pro-B cells	Mouse	Lescale C. et al., Nat Com. 2016
	6943.2	NA	NA	v-Abl/Bcl2 immortalization of bone marrow pro-B cells	Mouse	Lescale C. et al., Nat Com. 2016
	15307	NA	NA	v-Abl/Bcl2 immortalization of bone marrow pro-B cells	Mouse	Lescale C. et al., Nat Com. 2016; Jacks, T. et al, Curr. Biol. 1994
Xrcc4-/-	Xr95-22	-316bp		CRISPR-CAS9 knockout	12095	Lescale C., Lenden Hasse H., et al. Cell Reports. 2016
	Xr95-23	-288bp		CRISPR-CAS9 knockout	12095	Lescale C., Lenden Hasse H., et al. Cell Reports. 2016
	Xr95-50	-484bp	-514bp	CRISPR-CAS9 knockout	12095	Lescale C., Lenden Hasse H., et al. Cell Reports. 2016
Xrcc4-/- p53-/-	Xr15307-3	-422bp		CRISPR-CAS9 knockout	15307	This study
	Xr15307-11	-255bp		CRISPR-CAS9 knockout	15307	This study
Polq-/- p53-/-	Polq-2-307	-189bp	-183bp	CRISPR-CAS9 knockout	15307	This study
	Polq-5-307	-323bp		CRISPR-CAS9 knockout	15307	This study
Xrcc4-/- Polq-/- p53-/-	Polq-2-307-Xr25	-4bp		CRISPR-CAS9 knockout	Polq-2-307	This study
	Polq-2-307-Xr211	-324bp		CRISPR-CAS9 knockout	Polq-2-307	This study
	Xr307-3-Polq35	-240bp	-265bp	CRISPR-CAS9 knockout	Xr15307-3	This study
Rag2-/- p53-/-	17585	NA	NA	v-Abl/Bcl2 immortalization of bone marrow pro-B cells	Mouse	This study
	17587	NA	NA	v-Abl/Bcl2 immortalization of bone marrow pro-B cells	Mouse	This study
Rag2-/- Xrcc4-/- p53-/-	Xr-17585-17	-288bp		CRISPR-CAS9 knockout	17585	This study
	Xr-17585-18	-288bp		CRISPR-CAS9 knockout	17585	This study
Rag2-/- Polq-/- p53-/-	Polq-17587-8	-183bp		CRISPR-CAS9 knockout	17587	This study
	Polq-17587-13	-202bp		CRISPR-CAS9 knockout	17587	This study
	Polq-17587-22	-183bp		CRISPR-CAS9 knockout	17587	This study
Rag2-/- Xrcc4-/- Polq-/- p53-/-	X-P-87-4-4-X-3	-288bp		CRISPR-CAS9 knockout	Polq-17587-4	This study
	X-P-87-4-4-X-14	-288bp		CRISPR-CAS9 knockout	Polq-17587-4	This study
Parp1-/- p53-/-	6284	NA	NA	v-Abl/Bcl2 immortalization of bone marrow pro-B cells	Mouse	Galindo-Campos M.A., et al. Cell Death & Differentiation. 2019
	6286	NA	NA	v-Abl/Bcl2 immortalization of bone marrow pro-B cells	Mouse	Galindo-Campos M.A., et al. Cell Death & Differentiation. 2019
Parp1-/- Xrcc4-/- p53-/-	Xr-P1P2Fp53-2	-764bp		CRISPR-CAS9 knockout	6284	This study
	Xr-P1P2Fp53-3	-214bp		CRISPR-CAS9 knockout	6284	This study
	Xr-P1P2Fp53-4	-299bp		CRISPR-CAS9 knockout	6284	This study

Supplementary Table 2

List of primers and gRNAs used in the study

A. CRISPR oligonucleotide and PCR primer sequences. *Linker sequences for cloning into BsmBI-disgested MLM3636 are underlined; **gRNA score and predicted off-targets are determined using the online CRISPR Design tool http://crispr.mit.edu/ website from the Zhang lab.									
**Oligonucleotide name**	**Oligo sequence (5'->3')***	**sgRNA target coordinate (mm10)**	**Score****	**Deletion**	**Primer name**	**Primer sequence (5′-> 3′)**	**Expected PCR size wild type allele**	**Expected PCR size CRISPR/Cas9 edited allele**	**Deletion size**
sgXrcc4_1_A	ACA CCG GAA TGT ATA ACA GGA GAC GGG	chr13: 90,062,346–90,062,365	79	Part of the XRCC4 functional core region	pcr Xrcc4 1F	GGC TGA CAG TCT GAG GCT AT	1078 bp	790 bp	288 bps
sgXrcc4_1_B	AAA ACC CGT CTC CTG TTA TAC ATT CCG				pcr Xrcc4 1R	GCC TCC ATG TCA GTA CTG GT			
sgXrcc4_2_A	ACA CCG GCC GAG ACT CCT TAG AAA AGG	chr13: 90,062,044–90,062,063	65		pcr Xrcc4 2F	CCC TCA CAG AAA CAC AAC TCA	1171 bp	883 bp	
sgXrcc4_2_B	AAA ACC TTT TCT AAG GAG TCT CGG CCG				pcr Xrcc4 2R	CAA GGA GGT GGC CAC TAG TT			
sgPolq_1_A	ACA CCG TTG GGG TTC TCC CGC GCG GG G	chr16: 37,011,828-37,011,847	96	Polq first exon	pcr Polq 1F	CCG TCC CTC ACC ACT CTA TG	580 bp	398 bp	182 bps
sgPolq_1_B	AAA ACC CCG CGC GGG AGA ACC CCA ACG				pcr Polq 1R	AAC GCC CAT ATC TAC TGG GG			
sgPolq_2_A	ACA CCG CTG AGT CCA CCT CCG GGG CTG	chr16: 37,012,011-37,012,030	82		pcr Polq 2F	GAA GCC AGA TAT CAG CGC G	572 bp	390 bp	
sgPolq_2_B	AAA ACA GCC CCG GAG GTG GAC TCA GCG				pcr Polq 2R	AAC ACC ATC AGC TGT GCA TG			
**B. TOPO TA cloning**									
T7 primer	TAA TAC GAC TCA CTA TAG GG								
SP6 primer	ATT TAG GTG ACA CTA TAG								
**C. Nested-PCR for *IgkJ4* and *IgkV10-95* coding join**									
JK4-pa	CGC TCA GCT TTC ACA CTG ACT								
JK4-pb	CAG GTT GCC AGG AAT GGC TC								
V10-95-pa	TTC CTT GGT CTC CTG TTG CT								
V10-95-pb	TCT TGT TGA CTG GCG TTT TG								
IMR42	CTAGGCCACAG AATTGAAAGATCT								
IMR43	GTAGGTGGAAATTCTAGCATGA TGC								
**D. LAM-HTGTS oligos**									
Biotin-*IgkJ4*	Biotin-CGC TCA GCT TTC ACA CTG ACT								
Biotin-cmyc	Biotin-TGA TGT TGG GCT AGC GCA								
Adapter-upper-6N	GCG ACT ATA GGG CAC GCG TGG NNNNNN -NH2								
Adapter-lower-NH2	/5Phos/CCA CGC GTG CCC TAT AGT CGC-NH2								
P5-I5	AAT GAT ACG GCG ACC ACC GAG ATC TAC ACT CTT TCC CTA CAC GAC GCT CTT CCG ATC T								
P7-I7	CAA GCA GAA GAC GGC ATA CGA GAT CGG TCT CGG CAT TCC TGC TGA ACC GCT CTT C								
I5-Red (IgkJ4)	TAC ACT CTT TCC CTA CAC GAC GCT CTT CCG ATC T(6nts barcode)CAG GTT GCC AGG AAT GGC TC								
I5-Red (c-myc)	TAC ACT CTT TCC CTA CAC GAC GCT CTT CCG ATC T(6nts barcode)AGG GAT GTG ACC GAT TCG TTG								
I5-Red (CMV3)	TAC ACT CTT TCC CTA CAC GAC GCT CTT CCG ATC T(6nts barcode)GT ACG GTG GGA GGT CTA TA								
I7-Blue	CTC GGC ATT CCT GCT GAA CCG CTC TTC CGA TCT GAC TAT AGG GCA CGC GTG G								
**E. RT-PCR primers**									
Polq forward	TGGCTATATGGGCAGCACCT								
Polq reverse	CAGAGCAATGCCCTTGGATTT								
β-Actin forward	GGCTGTATTCCCCTCCATCG								
β-Actin reverse	CCAGTTGGTAACAATGCCATGT								
**F. sgRNA oligonucleotides and primers for Cas9-induced translocation assays**									
gRNA-Abl	GTTAACTTGAGGACCGGGAT								
gRNA-Bcr	GTATCACCCTCCTTTAGCGT								
Abl breakpoint PCR F	ATTCTGCAACTTTGGCCTGGTG								
Abl breakpoint PCR R	ACACCTGGAGTAAGCAGGACAC								
Bcr breakpoint PCR F	TGGGAGTCTTAAAGGCAGGAGC								
Bcr breakpoint PCR R	GTCAGGAGAGGCAAGTCCAAGT								
Bcr-Abl breakpoint direct PCR F	GCATTTCTAACCCGAAGTTCC								
Bcr-Abl breakpoint direct PCR R	GCTCATTTGTTACCATGACCAG								

Supplementary Table 3

A ABLki WT pro-B cells (*12095+12096*)

	IgkV$_{10-95}$ coding end / Ig*kJ$_4$* coding end	# frequency
Ref:	TCAGCAGTATAGTAAGCTTCCTCC ATTCACGTTCGGCTCGGGGACAAAG	
1.	TCAGCAGTATAGTAAGCTTC**** ****ACGTTCGGCTCGGGGACAAAG	5/31
2.	TCAGCAGTATAGTAAGCT****** ATTCACGTTCGGCTCGGGGACAAAG	3/31
3.	TCAGCAGTATAGTAAGCTTCC*** ATTCACGTTCGGCTCGGGGACAAAG	3/31
4.	TCAGCAGTATAGTAAGCTTC*** ****ACGTTCGGCTCGGGGACAAAG	2/31
5.	TCAGCAGTATAGTAAGCTTCCTCTTTTCACGTTCGGCTCGGGGACAAAG	2/31
6.	TCAGCAGTATAGTAAGCTTCCTCC *TTCACGTTCGGCTCGGGGACAAAG	2/31
7.	TCAGCAGTATAGTAAGCTTCCTC* ATTCACGTTCGGCTCGGGGACAAAG	1/31
8.	TCAGCAGTATAGTAAGCTTCCTC* ****ACGTTCGGCTCGGGGACAAAG	1/31
9.	TCAGCAGTATAGTAAGCTTATATTCACGTTCGGCTCGGGGACAAAG	1/31
10.	TCAGCAGTATAGTAAGCTTCC*** ATTCACGTTCGGCTCGGGGACAAAG	1/31
11.	TCAGCAGTATAGTAAGCTTCCAGTCACGTTCGGCTCGGGGACAAAG.	1/31
12.	TATAGTAAGCTTCCTCCGAAGTCACGTTCGGCTCGGGGACAAAG	1/31
13.	TCAGCAGTATAGTAAGCTTCCTCCAGTTCACGTTCGGCTCGGGGACAAAG	1/31
14.	TCAGCAGTATAGTAAGCTTC**** ATTCACGTTCGGCTCGGGGACAAAG	1/31
15.	TCAGCAGTATAGTAAGCTTCCCCC *TTCACGTTCGGCTCGGGGACAAAG	1/31
16.	TCAGCAGTATAGTAAGCCCGATTCACGTTCGGCTCGGGGACAAAG.	1/31
17.	TCAGCAGTATAGTAAGCTTCCT** ATTCACGTTCGGCTCGGGGACAAAG	1/31
18.	TCAGCAGTATAGTAAGCTTCCTCC *TTCACGTTCGGCTCGGGGACAAAG	1/31
19.	TCAGCAGTATAGTAAGCTTCCT** ATTCACGTTCGGCTCGGGGACAAAG	1/31
20.	TCAGCAGTATAGTAAGC******* ATTCACGTTCGGCTCGGGGACAAAG	1/31

B Released WT pro-B cells (*12095+12096*)

	IgkV$_{10-95}$ coding end / *IgkJ$_4$* coding end	# frequency
Ref:	TCAGCAGTATAGTAAGCTTCCTCC ATTCACGTTCGGCTCGGGGACAAA	
1.	TCAGCAGTATAGTAAGCTTCC*** ATTCACGTTCGGCTCGGGGACAAAG	5/24
2.	TCAGCAGTATAGTAAGCTTCCTC* ****ACGTTCGGCTCGGGGACAAAG	4/24
3.	TCAGCAGTGTAGTAAGCTT***** ATTCACGTTCGGCTCGGGGACAAAG	2/24
4.	TCAGCAGTATAGTAAGCTTCCTCC ****ACGTTCGGCTCGGGGACAAAG	2/24
5.	TCAGCAGTATAGTAAGCTTCCT** ***CACGTTCGGAGGGGGGACCAAG	2/24
6.	TCAGCAGTATAGTAAGCTTCC*** *****CGTTCGGCTCGGGGACAAAG	1/24
7.	TCAGCAGTATAGTAAGCTTCCTTATTCACGTTCGGCTCGGGGACAAAG	1/24
8.	TCAGCAGTATAGTAAGCTTCCTC* **TCACGTTCGGCTCGGGGACAAAG	1/24
9.	TCAGCAGTATAGTAAGCTTACATTCACGTTCGGCTCGGGGACAAAG	1/24
10.	TCAGCAGTATAGTAAGCTTC**** **TCACGTTCGGCTCGGGGACAAAG	1/24
11.	TCAGCAGTATAGTAAGCT****** ATTCACGTTCGGCTCGGGGACAAAG	1/24
12.	TCAGCAGTATAGTAAGCTTC**** ***CATTCACGTTCGGCTCGGGGACA	1/24
13.	TCAGCAGTATAGTAAGCTTCCTCC **TCACGTTCGGCTCGGGGACAAAG	1/24
14.	TCAGCAGTATAGTAAGCTTCCATATTCACGTTCGGCTCGGGGACAAAG	1/24

TTC: Micro-homology
AT : Insertion
** : Resection

C ABLki *Xrcc4* $^{-/-}$ *p53* $^{-/-}$ pro-B cells *(Xr15307-3)*

IgkV$_{10\text{-}95}$ coding end / *IgkJ*$_4$ coding end	Frequency
CCTGAAGATATTGCCACTTACTATTGTCAGCAGTATAGTAAGCTTCCTCC ATTCACGTTCGGCTCGGGGACAAAGTTGGAAATAAAACGTAAGTAGACTTTTGCTCATTTACTTGTG	
CCTGAAGATATTGCCACTT<u>ACT</u>***TTTGCTCATTTACTTGTG	9/9

D Released *Xrcc4* $^{-/-}$ *p53* $^{-/-}$ pro-B cells *(Xr15307-3)*

IgkV$_{10\text{-}95}$ coding end ... *IgkJs* coding end	Frequency
1.AGGAGTCCCATCAAGGTTCAG<u>TGGC</u> ** (95 bp)s ** .... ** (112 bps *J4)* ** TAAATGAGCCATTCCTGGCAA	4/36
2. AAGGTTCAGTGGCAGTGGGT<u>CTGGG</u> ** (83 bps) **........ ** (177 bps *J1)* ** AATAGGCTAGACATGTTCTCTGG	3/36
3.GGTTCAGTGGCAGTGGGTCTGGG<u>G</u> ** (82 bps) ** .... ** (86 bps *J4)* **ACGTTTTGGTTCTGTTTGGGTA	2/36
4.GGTTCAGTGGCAGTGGG ** (89 bps) **...*GTTTAACAGTTCCATCTGGTTTCG*...** (117 bps *J1)* ** CTCTGAAACCAGATTCT	2/36
5.CACCATCAGCAACCTGGAACCTGA<u>AGAT</u> ** (41 bps) ** .... ** (518 bps *J2)* ** TTGTGGGAGAAATGAGAAA	2/36
6.CATCAAGGTTCAGTGGCAGTGG<u>GTCT</u> ** (86 bps) ** .... ** (34 bps *J4)* ** ACTTACGTTTTATTTCCAAC	1/36
7.GATATGATATCCAGATGACACAG<u>ACT</u> ** (266 bps) ** .... ** (168 bps *J2)* ** GTCCCACAAGAGGTTGGAATG	1/36
8.AGTTTACACTCAGGAGTCCCATCAAT ** (106 bps) ** .... ** (235 bps *J2)* ** AACTAGGGGAAGAGGGATA	1/36
9. GATATGATATCCAGATGAC<u>ACAGACT</u> ** (266 bps) ** .... ** (500 bps *J2* ** GACCTCATGTCAGATTTGTGG	1/36
10. ATCAAGGTTCAGTGGCAGTGGGT<u>C</u> ** (88 bps) ** .... ** (9 bps *J4)* ** GGCTCGGGGACAAAGTTGGAAA	1/36
11. ATTGTCAGCAGTATAGTAAGCTTC ** (4 bps) ** .... ** (151 bps *J1)* ** TGCTCATTTACTTGTGACGTT	1/36
12. TTATTCTCTCACCATCAGCAACCT<u>GGA</u> ** (51 bps) ** .... ** (18 bps *J2)* ** CCAAGCTGGAAATAAAAC	1/36
13. ACCATCAGCAACCTGGAA<u>C</u> ** (49 bps) ** ....** (25 bps *J1)* ** AAACGTAAGTAGAATCCAAA	1/36
14. ATCAGCAACCTGGAACCTG<u>AA</u> ** (46 bps) **.........** (95 bps *J1)* ** AATGATGTATAAAATCTTACT	1/36
15. AGTCACCATCAGTTGCAGGG<u>CAAA</u> ** (211 bps) ** .... ** (407 bps *J2)* ** ACTTAAATAGAAGAGAACCAAA	1/36
16. CACCATCAGTGGCAGTGGGTCTGGG<u>G</u> ** (83 bps) ** ....** (66 bps *J4)* ** ACGTTTTGGTTCTGTTTGGGTAA	1/36
17. AGGTTCAGTGGCAGTGGGTCTGG ** (84 bps) ** ...AACAGA... ** (421 bps *J2)* ** GATAAATGAACTATTC	1/36
18. ACTATTGTCAGCAGTATAGTAAGCTTC ** (4 bps) ** .... ** (52 bps *J4)* ** TGCTCATTTACTTGTGACGTTTT	1/36
19. GGATTACACTCAGGAGTCCCATCA<u>AGG</u> ** (104 bps) **........** (211 bps *J2)* ** CTTTCTAAACCAAAGTAACTAAA	1/36
20. TCAGGAGTCCCATCAAGGTTCAGTG<u>G</u> ** (96 bps) **........** (10 bps *J4)* ** GCTCGGGGACAAAGTTGGAAACAA	1/36
21. GTGGCAGTGGGTCT ** (86 bps ** ...ACTTACGTTTTATTTCCAACTTTGTCCCCGAGCCG... ** (32 bps *J4)* ** AACGTAAGTAGACTT	1/36
22. ACAGACTACTTCCTCCCTGT<u>CTGCC</u> ** (248 bps) ** .... ** (274 bps *J1)* ** CTAGACAAACCTTTACTCGGTGCT	1/36
23. CCATCAAGGTTCAGTGGCAGTGGGT<u>C</u> ** (87 bps) ** ....** (8 bps *J4)* ** GGCTCGGGGACAAAGTTGG	1/36
24. TCTCACCATCAGCAACCTGGAACC ** (48 bps) ** ...AGA... ** (97 bps *J4)* ** AATTTGTGACATTTGGATAAATGA	1/36
25. ACAGACTACTTCCTCCCTGTCT<u>GCCT</u> ** (246 bps) ** .... ** (582 bps *J2)* ** CAGGCAGGTTTTTGTAAAGGGGGGC	1/36
26. CAAGGTTCAGTGGCAGTGGGTCTGG ** (84 bps) ** ...*AACAGA... ** (423 bps J2) *** GATAAATGAACTATTCCTTGTAACC	1/36
27. AACCTGGAACCTGAAGATATTGCCACTTACT ** (28 bps) ** ...TTTGCCAACAGG... ** (228 bps *J2)* ** GTAACTAAACTAGGGGAAG	1/36
28. GGGCAAGTGAGGACATTAGCACTT<u>ATTT</u> ** (189 bps) ** ....** (193 bps *J2)* ** TCAGGCTAAATTTTAGG	1/36

Released *Xrcc4* $^{-/-}$ *p53* $^{-/-}$ pro-B cells *(Xr15307-11)*

IgkV$_{10\text{-}95}$ coding end ... *IgkJs* coding end	Frequency
1. AGGAGTCCCATCAAGGTTCAG<u>TGGC</u> ** (95 bps) **........** (112 bps *J4)* ** TAAATGAGCCATTCCTGGCAA	7/34
2. ACTCCTGATCTATTACACA<u>TC</u> ** (132 bps) **........** (13 bps *J4)* **GGGGACAAAGTTGGAAATAAAACG	6/34
3. GCAGTGGGTCTGGGGCAGATTATTC<u>TCTC</u> ** (68 bps) **........** (552 bps *J2)* ** TGAACTTAGCCTATCT	4/34
4. GCGTTTTGAAGGGGTCCTTTCTT<u>TTC</u> ** (339 bps) **........ **(1 bp *J4)* ** ACGTTCGGCTCGGGGACAA	4/34
5. CTTGTTGACTGGCGTTTTGAAG<u>GGGT</u> ** (350 bps) **........** (267 bps *J1)* ** AGCCTGCCCTAGACAAACC	3/34
6. TGAAGATATTGCCACTTACTATTG<u>TC</u> ** (22 bps) **........** (60 bps *J1)* ** TTCCGTTGTCTATGTCTGT	1/34
7. GATGGAACTGTTAAACTCCTGAT<u>C</u> ** (143 bps) **........** (107 bps *J4)* ** ATTTTGGCTAAATGAGCCATTCC	1/34
8. CATCAAGGTTCAGTGGCAGTGG<u>G</u> ** (89 bps) **........** (335 bps *J2)* ** AGAAATGAGAAAGGAACAGTTTT	1/34
9. CAAGGTTCAGTGGCAGTGGGTCT<u>GG</u> ** (84 bps) **........** (152 bps *J2)* ** TGTTAAGGAGGGAAAACTGTC	1/34
10. TCTTGTTGACTGG<u>CGTTTTG</u> ** (357 bps) **........** (68 bps *J4)* ** GTTCTGTTTGGGTAACTTGTGTGA	1/34
11. GATGGAACTGTTAAACTCCTGATC ** (143 bps) ** ....*G.... ** (109 bps J4) *** TTTTGGCTAAATGAGCCATTCC	1/34
12. CAAGGTTCAGTGGCAGTGGGT<u>CTGG</u> ** (84 bps) **........** (131 bps *J1)* ** CACTCTCCAAGGCAAAGAT	1/34
13. ACAGAGTCACCATCAGTTGCAG<u>GG</u> ** (214 bps) **........**(187 bps *J2)* **AATGATTTTCAGGCTAAATTTT	1/34
14. GATTACACTCAGGAGTCCCATC<u>AAGG</u> ** (104 bps) **........** (158 bps *J2)* ****AGGGAAAACTGTCCCACAA	1/34
15. CTCAGGAGTCCCATCAAGGTTCAG<u>TGGC</u> ** (13 bps) **........** (112 bps *J4)* **TAAATGAGCCATTCCTGGC	1/34

Supplementary Table 3.
Related to Figure 1.

IgkV$_{10\text{–}95}$-*J*$_4$ coding joins sequences from ABLki WT (A), released WT (B), ABLki *Xrcc4*$^{-/-}$ *p53*$^{-/-}$ (C) and released *Xrcc4*$^{-/-}$ *p53*$^{-/-}$ (D) *v-Abl* proB *cells.* The reference sequence for 5' and 3' ends of *IgkV*$_{10\text{–}95}$-*J*$_4$ coding join is shown at the top (green for *IgkV*$_{10\text{-}95}$ and red for *IgkJs* coding end respectively). * indicate the number of resected base pairs. For long resection, the number of resected nucleotides is indicated in brackets. Microhomologies are underlined. Insertions are shown in purple.

Supplementary Table 4

A

% All	WT ABLKi (#4)	WT Re (#5)	Xrcc4-/- ABLKi (#4)	p53-/- ABLKi (#3)	p53-/- Re (#3)	Xrcc4-/- p53-/- ABLKi (#3)	Xrcc4-/- p53-/- Re (#6)
Total #	4855815	6196042	2664685	2045431	2162548	1692118	4770380
Germline # (%)	4 242 318 (87.36)	5 973 012 (96.4)	2 664 148 (99.99)	1 872 047 (91.52)	1 568 666 (72.53)	1 691 427 (99.95)	4 589 255 (96.20)
Total re-arrangements	613 497 (12.63)	223 030 (3.6)	515 (0.02)	173 384 (8.47)	593 882 (12.63)	691 (0.041)	181 125 (3.797)
IgK re-arrangement # (%)	612 718 (12.61)	221 486 (3.57)	431 (0.016)	172 054 (8.41)	590 228 (27.29)	484 (0.029)	162 649 (3.409)
JK4-AgR # (%)	129 (0.003)	96 (0.002)	3 (0.0000)	40 (0.002)	776 (0.036)	4 (0.0002)	6 439 (0.134)
JK4-IgH/IgL # (%)	127 (0.003)	96 (0.002)	3 (0.0000)	40 (0.002)	765 (0.035)	4 (0.0002)	6 311 (0.132)
# JK4-else # (%)	650 (0.013)	1 449(0.023)	104 (0.004)	1 290(0.063)	2 878 (0.133)	205 (0.012)	11 985 (0.251)

B

% All	WT NT (#2)
Total #	501850
Germline # (%)	501 721 (99.97)
Total re-arrangements # (%)	129 (0.0257)
IgK re-arrangement # (%)	39 (0.007)
JK4-AgR # (%)	76 (0.0151)
JK4-IgH/IgL # (%)	76 (0.0151)
# JK4-else # (%)	14 (0.0027)

C

	NT	ABLKi	Re
Total #	11 352 700	7 386 173	14 580 394
Reads with *IgK*	6 958 435	2 177 414	4 999 955
Vk-Jk joins # (%)	221 (0.003)	63 (0.002)	5 882 (0.117)

Supplementary Table 4.
Related to Figure 1.
(A) Summary of LAM-HTGTS sequencing data analysis using *IgkJ4* bait in *v-Abl* pro-B cell lines. Upper table: number (#) of germline and rearrangement reads for ABLki and released (Re) cell lines. Sequenced cell lines: *WT (12095, 12096); Xrcc4$^{-/-}$ (Xr95-22, Xr95-23, Xr95-50); p53$^{-/-}$ (15307, 6920.1); Xrcc4$^{-/-}$ p53$^{-/-}$ (Xr15307-3, Xr15307-11).*
(B) Number (#) of germline and rearrangement reads for untreated cell lines. Analysis steps are explained in Figure S5. **(C)** Summary of Agilent captured sequencing data analysis in *Xrcc4* $^{-/-}$ *p53* $^{-/-}$ pro-B cell line. Percentages correspond to the number of reads relative to total number of reads with *Igk* locus sequence. Sequenced cell lines: *Xrcc4$^{-/-}$ p53$^{-/-}$ (Xr15307-3).*

Supplementary Table 5

Sequence analysis of *Bcr-Abl* chromosomal translocations

Sample			Resection Length (bps)		microhomology		Insertion	
	# Sequence	Frequency	BCR end	ABL end	Length (bps)	Sequence	Length (bps)	Sequence
Cycling Rag2-/- p53-/-	1	5/60	67	484	4	AGTG		
	2	5/60	318	170	2	TC		
	3	4/60	67	444	2	TG		
	4	3/60	91	1	4	ATTG		
	5	3/60	1	4			14	TATCCTTTGAAATG
	6	3/60	16	4	1	G		
	7	3/60	324	68	3	GGA		
	8	3/60	69	22	5	GAGAG		
	9	2/60	0	0				
	10	2/60	44	47	4	TGCT		
	11	2/60	16	7	2	GG		
	12	2/60	24	49	3	CTG		
	13	2/60	0	0			1	C
	14	1/60	21	20			5	GAAGG
	15	1/60	285	23	1	T		
	16	1/60	2	0			2	CG
	17	1/60	73	47	4	TGCT		
	18	1/60	15	18	3	GGT		
	19	1/60	290	23			4	GTGC
	20	1/60	2	2	2	TT		
	21	1/60	0	1				
	22	1/60	102	127			1	C
	23	1/60	46	50	2	TG		
	24	1/60	324	76			5	CGGTG
	25	1/60	64	37	4	GAGG		
	26	1/60	105	121	4	CAGC		
	27	1/60	39	92	3	CTC		
	28	1/60	3	2			3	AAC
	29	1/60	0	56			12	GTCTTTGCATCT
	30	1/60	79	72	2	GG		
	31	1/60	116	57	6	AGGCAG		
	32	1/60	72	25			1	A
	33	1/60	24	46	3	CTG		
	34	1/60	22	114	6	TCTGTC		

Cycling Rag2 -/- Xrcc4 -/- p53 -/-	1	3/83	131	7	2	GG		
	2	2/83	2	317				
	3	2/83	44	47	4	TGCT		
	4	2/83	91	1	4	ATTG		
	5	2/83	335	17			4	GAAA
	6	2/83	7	475			3	TTG
	7	2/83	211	20				
	8	2/83	16	17	2	GG		
	9	2/83	15	18	3	ACC		
	10	2/83	21	309	3	TCA		
	11	2/83	61	80	2	TG		
	12	2/83	16	29	3	AGG		
	13	2/83	312	66	5	TCCTG		
	14	2/83	16	31	2	CC		
	15	1/83	16	38	3	AGG		
	16	1/83	3	31			5	GAGTT
	17	1/83	24	17	1	G		
	18	1/83	58	92	2	CT		
	19	1/83	4	0			4	GGGT
	20	1/83	12	114	2	TC		
	21	1/83	0	17			2	GA
	22	1/83	2	2	2	TT		
	23	1/83	10	2			3	ATC
	24	1/83	16	16	2	GG		
	25	1/83	3	112	3	CTT		
	26	1/83	259	9				
	27	1/83	338	95	4	CCTA		
	28	1/83	273	10			4	GATG
	29	1/83	18	25	1	A		
	30	1/83	243	10	2	CA		
	31	1/83	38	226			8	CTTTAGTC
	32	1/83	26	226	4	GATT		
	33	1/83	26	0			2	CC
	34	1/83	7	45	3	AGG		
	35	1/83	26	226	4	AATC		
	36	1/83	22	54	2	TC		
	37	1/83	313	84	3	CCT		
	38	1/83	18	29			2	TT
	39	1/83	24	31	1	G		
	40	1/83	61	80	2	TG		
	41	1/83	22	31			230	TTGAGCAACAAAAATTTAAGTACATTCT<br>GCAACTTTGGCCTGGTGAGCTGTGATC<br>ATTCTTACAGTTGATGAGTATGGCTGAC<br>TATGAAATGAAGAAAGTAACTTGGCAC<br>TGATGCACTGTGAGTCAGTTTTGACATC<br>AGCCTGGACTGGGAATTGGCTGGAAG<br>GTTTTGTTGCTACACTCCATGTCTCCATC<br>CAGAGGCACACATGAGCAATACTTCCCT<br>TTGGGCTTT
	42	1/83	32	21			26	CTCTCACCTTGGGCCCTGGGAGACAC
	43	1/83	79	83	4	AAGG		
	44	1/83	72	171				
	45	1/83	16	4	2	GG		
	46	1/83	157	107	1	G		
	47	1/83	17	8	1	C		
	48	1/83	64	57	3	AGG		
	49	1/83	15	10	1	T		
	50	1/83	4	18			182	CAAGTTAACAGCCAAAGCCCAAAGGGA<br>AGTATTGCTCATGTGTGCCTCTGGATGG<br>AGACATGGAGTGTAGCAACAAAACCTT<br>CCAGCCAATTCCCAGTCCAGGCTGATGT<br>CAAAACTGACTCACAGTGCATCAGTGCC<br>AAGTTACTTTCTTCATTTCATAGTCAGCC<br>ATACTCATCAACTGC
	51	1/83	7	22			204	TCTTACAGTTGATGAGTATGGCTGACTA<br>TGAAATGAAGAAAGTAACTTGGCACTG [illegible]
	52	1/83	50	235	1	G		
	53	1/83	43	100			10	CACTCCCTAT
	54	1/83	49	235	2	GC		
	55	1/83	10	30			378	TGATGGCTGTGGTGTTCTTACTTCTCCA<br>CTCCAGTTCCTCTACACCCTGTGACTTGT<br>TGTTTCGGGGTGCTGGGAAAATTGAGT<br>TCTGGATGTGAGGTAATACAAAGGCTG<br>TATTTAGTTAAATGCGGTTTTGTTATTTT<br>AATAATCTGAACTGTGGGTGAGGGTCT<br>GAGTGATAAGAGGGATGCAGTGGCGT<br>TCAGCAGGTTTACATTGCGGGAAATAA<br>GACTTTTCCTCCAAGCTCACATTATGAG<br>GAACTCACGAAACAGTTACAGACTGACT<br>TGAGAATATTCTGAGCATGACCTCATCA<br>GCTGGAACAGAAAGGAGCTAAAACATG<br>AAGTCCTCTCCTTTGCAAAGGGTCTGTT<br>TTACAGATCTGCGCATTGC
	56	1/83	303	56	2	CA		
	57	1/83	29	11			11	GTCTGTCAAAA
	58	1/83	49	40	2	GC		
	59	1/83	324	17			1	A
	60	1/83	12	412			5	GCCCT
	61	1/83	49	235	2	GC		
	62	1/83	4	412	3	CCT		
	63	1/83	76	8	1	A		
	64	1/83	4	314	4	TCCT		
	65	1/83	256	24			30	CACCCTACCCTCTCTCTCAACCCCACCCCA
	66	1/83	3	15			1	A
	67	1/83	48	48	3	GCT		
	68	1/83	191	25				

G1 Rag2-/- p53-/-	1	3/3	381	517	2	CC		
G1 Rag2-/- Xrcc4-/- p53-/-	1	3/8	313	84	3	AGG		
	2	3/8	13	9	2	AT		
	3	2/8	62	67	5	GTCCT		
Released Rag2-/- p53-/-	1	6/9	0	0				
	2	3/9	1	0	1	A		
Released Rag2-/- Xrcc4-/- p53-/-	1	4/84	156	255	1	A		
	2	4/84	62	286			1	C
	3	4/84	57	32	4	GCTT		
	4	4/84	373	20	2	TT		
	5	4/84	381	412	2	CC		
	6	3/84	44	47	4	TGCT		
	7	2/84	180	42	5	GGATG		
	8	2/84	58	163			1	G
	9	2/84	45	369	3	TGC		
	10	2/84	52	49	2	CT		
	11	2/84	46	273				
	12	2/84	48	221			1	A
	13	2/84	35	463			4	AAGT
	14	2/84	38	442	3	TCT		
	15	2/84	99	48	5	GCTGG		
	16	2/84	29	143	3	TCA		
	17	2/84	39	412	1	C		
	18	1/84	64	17	2	GG		
	19	1/84	15	72	3	ACC		
	20	1/84	48	46	2	CT		
	21	1/84	46	52	1	C		
	22	1/84	24	15	2	TG		
	23	1/84	11	48			4	TTTG
	24	1/84	15	7				
	25	1/84	58	48	3	GCT		
	26	1/84	12	6	1	C		
	27	1/84	15	18	3	GGT		
	28	1/84	49	52	2	GA		
	29	1/84	78	32			65	TAATATATTTAAAATGTCATTTAGAAGG<br>ACACAGAAGAAAGTGCTTGTGGCAGAG<br>AAGAGAGAGA
	30	1/84	31	19	2	GT		
	31	1/84	13	504	2	AT		
	32	1/84	49	26			4	CCTA
	33	1/84	15	13	3	ACC		
	34	1/84	18	335	5	CAAAA		
	35	1/84	46	439	3	TTG		
	36	1/84	15	25			4	GATA
	37	1/84	15	17			5	GGAGA
	38	1/84	46	32	5	GCTTG		
	39	1/84	16	4	2	GG		
	40	1/84	41	142	3	TTC		
	41	1/84	56	477	3	TTA		
	42	1/84	151	12	4	GGGT		
	43	1/84	48	48	3	GCT		
	44	1/84	49	25			3	CCT
	45	1/84	41	332	3	TTC		
	46	1/84	16	7	2	GG		
	47	1/84	293	455	2	CA		
	48	1/84	158	8	2	GA		
	49	1/84	7	256				
	50	1/84	323	11			6	AGTATA
	51	1/84	15	34				
	52	1/84	43	32	4	AAGC		
	53	1/84	15	18	3	GGT		
	54	1/84	57	31	5	AAGCT		
	55	1/84	161	255			4	CTCT
	56	1/84	21	456			745	CTGAACTGTGGGTGAGGGTCTGAGTG<br>ATAAGAGGGATGCAGTGGCGTTCAGCA<br>GGTTTACATTGTGGGAAATAAGACTTTT<br>CCTCCAAGCTCACATTATGAGGAACTCA<br>CGAAACAGTTACAGACTGACTTGAGAA<br>TATTCTGAGCATGACCTCATCAGCTGGA<br>ACAGAAAGGAGCTAAAACATGAAGTCC<br>TCTCCTTTGCAAAGGGTCTGTTTTACAG<br>ATCTGCGCATTGCGGAGATTCCTCTAGC<br>CAGAGGAAATCTCAAAGGGGCCACCAA<br>GGAAAGAAAGTTACTTTGGCAGGGCTT<br>AAGTCCTTGAAATGAGCACAGGAAATG<br>GGACTGTGTGTTTAGGGACATACCCTTT<br>CTGTTCCTCCCCAACGAGGAAGTGTCTC<br>CCAGGGCCCAAGGTGAGGACTGTGGG<br>AAGTGCTGTGCAGCTGGCTGCCCCACAC<br>TCCACGGGGACCTGGGGACCAGTGCCA<br>ACAGGAAGTCAGGAGCTTCCTGGGGAT<br>GTTTTTCCTGGAGGCAAATTCTACCGTC<br>TCCCTGAGACAGTGAAGTCTCCTTCCCCT<br>GGGTCTCAGGGCAGCCACTGGCTCCCCT<br>CATTCCTCACAGCGATCTAGCCCAGATAT<br>GCCCCATCCAAGGATCTTCTGTAAACAG<br>TCTTCAACAGTGACTCACAACCTTGAAA<br>AGGGATTTCCTTTTATTTTAATAAAAAG<br>GACCTTTAACATACTCAAACAGTTTTCCT

Supplementary Table 5.
Related to Figure 3.
TOPO cloning sequence analysis for PCR products of *Bcr-Abl* translocation breakpoint junctions including resection length from *Bcr* and *Abl* breakpoint sites and microhomology and insertion at breakpoint junctions. Each genotype represents two independent cell clones.

Supplementary Table 6

A

Genotype	Cell line + Treatment	Total metaphases analyzed	Total normal metaphases	Metaphases with chromosome breaks	Metaphases with translocations	aberrant metaphases	% Metaphases with chromosome breaks	% Metaphases with translocations	% aberrant metaphases
p53-/-	15307 NT *	130	122	4	4	8	3.1	3.1	6.2
	6920.1 NT	146	143	1	2	3	0.7	1.4	2.1
	**Total**	**276**	**265**	**5**	**6**	**11**	**1.8**	**2.2**	**4.0**
	**Mean (±SD)**						**1.9 (±1.7)**	**2.2 (±1.2)**	**4.1 (±2.9)**
	6920.1 Re *	250	241	7	2	9	2.8	0.8	3.6
	6943.2 Re *	251	235	8	8	16	3.2	3.2	6.4
	15307 Re *	244	224	7	13	20	2.9	5.3	8.2
	**Total**	**745**	**700**	**22**	**23**	**45**	**3.0**	**3.1**	**6.0**
	**Mean (±SD)**						**3.0 (±0.2)**	**3.3 (±2.3)**	**6.1 (±2.3)**
Xrcc4-/- p53-/-	Xr15307-3 NT	218	217	0	1	1	0.0	0.5	0.5
	Xr15307-11 NT	155	154	0	1	1	0.0	0.6	0.6
	**Total**	**373**	**371**	**0**	**2**	**2**	**0.0**	**0.5**	**0.5**
	**Mean (±SD)**						**0 (±0)**	**0.55 (±0.1)**	**0.55 (±0.1)**
	Xr15307-3 Re (1)	257	162	48	50	98	18.7	19.5	38.1
	Xr15307-3 Re (2)	178	116	32	30	62	18.0	16.9	34.8
	Xr15307-3 Re (3)	61	41	7	13	20	11.5	21.3	32.8
	Xr15307-11 Re (1)	226	159	35	36	71	15.5	15.9	31.4
	Xr15307-11 Re (2)	91	54	18	15	33	19.8	16.5	36.3
	**Total**	**813**	**532**	**140**	**144**	**284**	**17.2**	**17.7**	**34.9**
	**Mean (±SD)**						**16.7 (±3.3)**	**18 (±2.3)**	**34.7 (±2.7)**
Xrcc4-/- p53-/- + Xrcc4	Xr15307-3 + Xrcc4 NT	95	91	4	0	4	4.2	0.0	4.2
	Xr15307-11 + Xrcc4 NT	110	109	0	1	1	0	0.9	0.9
	**Total**	**205**	**200**	**4**	**1**	**5**	**2.0**	**0.5**	**2.4**
	**Mean (±SD)**						**2.1 (±3)**	**0.5 (±0.6)**	**2.6 (±2.3)**
	Xr15307-3 + Xrcc4 Re	218	196	13	9	22	6	4.2	10.2
	Xr15307-11 + Xrcc4 Re	279	266	9	4	13	3.2	1.4	4.6
	**Total**	**497**	**462**	**22**	**13**	**35**	**4.4**	**2.6**	**7.0**
	**Mean (±SD)**						**4.6 (±2)**	**2.8 (±2)**	**7.4 (±4)**
Polq-/- p53-/-	Polq-2-307 NT (1)	53	53	0	0	0	0.0	0.0	0.0
	Polq-2-307 NT (2)	108	107	0	1	1	0.0	0.9	0.9
	Polq-5-307 NT	115	114	1	0	1	0.9	0.0	0.9
	**Total**	**276**	**274**	**1**	**1**	**2**	**0.4**	**0.4**	**0.7**
	**Mean (±SD)**						**0.3 (±0.5)**	**0.3 (±0.5)**	**0.6 (±0.5)**
	Polq-2-307 Re (1)	41	39	1	2	3	2.4	4.9	7.3
	Polq-2-307 Re (2)	120	117	1	2	3	0.8	1.7	2.5
	Polq-5-307 Re	111	110	0	1	1	0.0	0.9	0.9
	**Total**	**272**	**266**	**2**	**5**	**7**	**0.7**	**1.8**	**2.6**
	**Mean (±SD)**						**1.1 (±1.2)**	**2.5 (±2.1)**	**3.6 (±3.3)**
Xrcc4-/- Polq-/- p53-/-	Polq-2-307-Xr25 NT	92	86	4	2	6	4.3	2.2	6.5
	Xr307-3-Polq35 NT	214	210	3	1	4	1.4	0.5	1.9
	**Total**	**306**	**296**	**7**	**3**	**10**	**2.3**	**1.0**	**3.3**
	**Mean (±SD)**						**2.9 (±2.1)**	**1.3 (±1.2)**	**4.2 (±3.3)**
	Polq-2-307-Xr25 Re (1)	58	19	38	2	40	65.5	3.4	69.0
	Polq-2-307-Xr25 Re (2)	99	23	75	1	76	75.8	1.0	76.8
	Xr307-3-Polq35 Re (1)	101	34	62	5	67	61.4	5.0	66.3
	Xr307-3-Polq35 Re (2)	115	23	91	1	92	79.1	0.9	80.0
	**Total**	**373**	**99**	**266**	**9**	**275**	**71.3**	**2.4**	**73.7**
	**Mean (±SD)**						**70.5 (±8.4)**	**2.6 (±2.0)**	**73 (±6.4)**
Xrcc4-/- Polq-/- p53-/- + Xrcc4	Polq-2-307-Xr25 + Xrcc4 NT	187	187	0	0	0	0.0	0.0	0.0
	Xr307-3-Polq35 + Xrcc4 NT	166	166	0	0	0	0.0	0.0	0.0
	**Total**	**353**	**353**	**0**	**0**	**0**	**0.0**	**0.0**	**0.0**
	**Mean (±SD)**						**0 (±0)**	**0 (±0)**	**0 (±0)**
	Polq-2-307-Xr25 + Xrcc4 Re	192	190	0	2	2	0.0	1.0	1.0
	Xr307-3-Polq35 + Xrcc4 Re	125	123	1	1	2	0.8	0.8	1.6
	**Total**	**317**	**313**	**1**	**3**	**4**	**0.3**	**0.9**	**1.3**
	**Mean (±SD)**						**0.4 (±0.6)**	**0.9 (±0.2)**	**1.3 (±0.4)**
Rag2-/- Xrcc4-/- Polq-/- p53-/-	X-P-87-4-4-X-3 NT	254	254	0	0	0	0	0	0
	X-P-87-4-4-X-14 NT	266	265	0	1	1	0	0.4	0.4
	X-P-87-4-4-X-16 NT	180	180	1	0	1	0.6	0	0.6
	**Total**	**700**	**699**	**1**	**1**	**2**	**0.1**	**0.1**	**0.3**
	**Mean (±SD)**						**0.2 (±0.3)**	**0.1 (±0.2)**	**0.3 (±0.3)**
	X-P-87-4-4-X-3 Re	330	327	1	2	3	0.3	0.6	0.9
	X-P-87-4-4-X-14 Re	172	170	2	0	2	1.2	0	1.2
	X-P-87-4-4-X-16 Re	290	289	1	0	1	0.3	0	0.3
	**Total**	**792**	**786**	**4**	**2**	**6**	**0.5**	**0.3**	**0.8**
	**Mean (±SD)**						**0.6 (±0.5)**	**0.2 (±0.3)**	**0.8 (±0.5)**
Xrcc4-/- Parp1-/- p53-/-	Xr-P1P2Fp53-2 NT	204	201	2	1	3	1.0	0.5	1.5
	Xr-P1P2Fp53-3 NT	90	90	0	0	0	0.0	0.0	0.0
	**Total**	**294**	**291**	**2**	**1**	**3**	**0.7**	**0.3**	**1.0**
	**Mean (±SD)**						**0.5 (±0.7)**	**0.3 (±0.3)**	**0.8 (±1)**
	Xr-P1P2Fp53-2 Re	111	69	25	17	42	22.5	15.3	37.8
	Xr-P1P2Fp53-3 Re	146	95	28	23	51	19.2	15.8	34.9
	**Total**	**257**	**164**	**53**	**40**	**93**	**20.6**	**15.6**	**36.2**
	**Mean (±SD)**						**20.9 (±2.4)**	**15.5 (±0.3)**	**36.4 (±2.1)**

B

Statistical analysis (two-sided Fisher exact test)		Normal vs Aberrant (p-value)	Level
Xrcc4-/- p53-/- Re ***vs***	Xrcc4-/- p53-/- NT	3.8E-51	***
	p53-/- Re	4.9E-47	***
	Xrcc4-/- p53-/- + Xrcc4 Re	7.9E-34	***
	Xrcc4-/- Parp1-/- p53-/- Re	0.708	NS
Xrcc4-/- Polq-/- p53-/- Re ***vs***	Xrcc4-/- Polq-/- p53-/- NT	3.17E-89	***
	Polq-/- p53-/- Re	1.02E-85	***
	Xrcc4-/- p53-/- Re	4.79E-35	***
	Rag2-/- Xrcc4-/- Polq-/- p53-/	9.8E-172	***
	Xrcc4-/- Polq-/- p53-/- + Xrcc4	2.60E-100	***

* $p < 0.05$

*** $p < 0.01$

C

Genotype	Cell line + Treatment	Total metaphases analyzed	Total normal metaphases	Metaphases with chromosome breaks	Metaphases with translocations	Total aberrant metaphases	% Metaphases with chromosome breaks	% Metaphases with translocations	% aberrant metaphases
Rag2-/- p53-/-	17585 NT	37	36	0	1	1	0	2.7	2.7
	17587 NT	37	37	0	0	0	0	0	0
	**Total**	**74**	**73**	**0**	**1**	**1**	**0**	**1.4**	**1.4**
	**Mean (±SD)**						**0 (±0)**	**1.4 (±1.9)**	**1.35 (±1.9)**
	17585 Re	126	126	0	0	0	0	0	0
	17587 Re	65	65	0	0	0	0	0	0
	**Total**	**191**	**191**	**0**	**0**	**0**	**0**	**0**	**0**
	**Mean (±SD)**						**0 (±0)**	**0 (±0)**	**0 (±0)**
Rag2-/- Polq-/- p53-/-	Polq-17587-8 NT	98	90	5	3	8	5.1	3.1	8.2
	Polq-17587-22 NT	90	83	4	3	7	4.4	3.3	7.8
	**Total**	**188**	**173**	**9**	**6**	**15**	**4.8**	**3.2**	**8.0**
	**Mean (±SD)**						**4.8 (±0.5)**	**3.2 (±0.2)**	**8 (±0.3)**
Rag2-/- Xrcc4-/- p53-/-	Xr17585-17 (1) NT	32	20	6	6	12	18.8	18.8	37.5
	Xr17585-17 (2) NT	64	49	7	8	15	10.9	12.5	23.4
	Xr17585-18 NT	92	64	10	18	28	10.9	19.6	30.4
	**Total**	**188**	**133**	**23**	**32**	**55**	**12.2**	**17.0**	**29.3**
	**Mean (±SD)**						**13.5 (±4.5)**	**16.9 (±3.9)**	**30.4 (±7)**
	Xr17585-17 Re	45	30	5	9	14	11.1	20.0	31.1
	Xr17585-18 Re	53	33	9	11	20	17.0	20.8	37.7
	**Total**	**98**	**63**	**14**	**20**	**34**	**14.3**	**20.4**	**34.7**
	**Mean (±SD)**						**14 (±4.2)**	**20.4 (±0.5)**	**34.4 (±4.7)**
Rag2-/- Xrcc4-/- Polq-/- p53-/-	X-P-87-4-4-X-14 NT	42	27	13	2	15	31.0	4.8	35.7
	X-P-87-4-4-X-3 NT	101	59	33	9	42	32.7	8.9	41.6
	**Total**	**143**	**86**	**46**	**11**	**57**	**32.2**	**7.7**	**39.9**
	**Mean (±SD)**						**31.8 (±1.2)**	**6.8 (±2.9)**	**38.6 (±4.2)**
	X-P-87-4-4-X-3 Re	12	4	8	0	8	66.7	0	66.7

D

**Statistical analysis (two-sided Fisher exact test)**		**Normal *vs* Aberrant**	**Break *vs* Others**	**Translocation *vs* Others**
Rag2-/- Xrcc4-/- p53-/- NT *vs*	Rag2-/- p53-/- NT	6.10E-05		
	Rag2-/- Polq-/- p53-/- NT	1.24E-07		
	Rag2-/- Xrcc4-/- Polq-/- p53-/	0.046	1.74E-05	0.013
Rag2-/- Polq-/- p53-/- NT *vs*	Rag2-/- p53-/- NT	0.046		
	Rag2-/- Xrcc4-/- Polq-/- p53-/	2.98E-12		
Rag2-/- Xrcc4-/- p53-/- Re *vs*	Rag2-/- p53-/- Re	5.68E-12		

Supplementary Table 6. Genomic instability at the *Igk and Bcr/Abl* loci in pro-B cells. Related to Figure 4.

(A) Number and percentage of aberrant metaphases harboring chromosomes breaks and/or translocations involving the *Igk* locus from pro-B cell lines of the indicated genotype and respective treatment. DNA FISH experiments were performed using probes centromeric (*Igk V*) and telomeric (*Igk C*) to the *Igk* locus plus specific paint for chromosome 6. NT = untreated cycling conditions; Re = ABLki treatment followed by release of the cells into the cell cycle. * Data from Lescale C. et al. Nature Communications 2016. **(B)** Statistical analysis using two-sided Fisher exact test. **(C)** Number and percentage of aberrant metaphases harboring chromosomes breaks and/or translocations involving the *Bcr* and *Abl* loci from pro-B cell lines of the indicated genotype and respective treatment. NT = untreated cycling conditions + Cas9/gRNA nucleofection; Re = ABLki treatment + Cas9/gRNA nuclofection in blocked cells followed by release of the cells into the cell cycle. DNA FISH experiments were performed using probes to the *Bcr* and *Abl* loci. **(D)** Statistical analysis using two-sided Fisher exact test.
