# Supplementary material for: Repair of G1 induced DNA double-strand breaks in S-G2/M by alternative NHEJ
Source: Nat Commun. 2020 Oct 16;11:5239. doi: 10.1038/s41467-020-19060-w (PMC7567796; doi:10.1038/s41467-020-19060-w)
Supplement: Supplementary file 3 — Reporting Summary [file 41467_2020_19060_MOESM3_ESM.pdf]

nature research

Corresponding author(s): Ludovic Deriano

Last updated by author(s): Sep 3, 2020

Reporting Summary

Nature Research wishes to improve the reproducibility of the work that we publish. This form provides structure for consistency and transparency in reporting. For further information on Nature Research policies, see our Editorial Policies and the Editorial Policy Checklist.

Statistics

For all statistical analyses, confirm that the following items are present in the figure legend, table legend, main text, or Methods section.

n/a	Confirmed	
☐	☒	The exact sample size (*n*) for each experimental group/condition, given as a discrete number and unit of measurement
☐	☒	A statement on whether measurements were taken from distinct samples or whether the same sample was measured repeatedly
☐	☒	The statistical test(s) used AND whether they are one- or two-sided<br>*Only common tests should be described solely by name; describe more complex techniques in the Methods section.*
☒	☐	A description of all covariates tested
☒	☐	A description of any assumptions or corrections, such as tests of normality and adjustment for multiple comparisons
☐	☒	A full description of the statistical parameters including central tendency (e.g. means) or other basic estimates (e.g. regression coefficient) AND variation (e.g. standard deviation) or associated estimates of uncertainty (e.g. confidence intervals)
☐	☒	For null hypothesis testing, the test statistic (e.g. *F*, *t*, *r*) with confidence intervals, effect sizes, degrees of freedom and *P* value noted<br>*Give P values as exact values whenever suitable.*
☒	☐	For Bayesian analysis, information on the choice of priors and Markov chain Monte Carlo settings
☐	☒	For hierarchical and complex designs, identification of the appropriate level for tests and full reporting of outcomes
☐	☒	Estimates of effect sizes (e.g. Cohen's *d*, Pearson's *r*), indicating how they were calculated

Our web collection on statistics for biologists contains articles on many of the points above.

Software and code

Policy information about availability of computer code

Data collection	Gel/membrane Imaging: Image Lab v6.0; Microscopy: Metafer 4 v3.11.3; Flow cytometry: BD LSR Fortessa, FACS Canto II, Cell Sorter FACSAria III, FACS Diva v8.0.1. Junctions visualization: IGV v2.4.5
Data analysis	BWA v0.7.4; samtools v0.1.19; ea-utils v1.1.2; circos v0.64; bedtools v2.17.0; Flowjo v10.4.2; Graph Prism v7.04; Image Lab v6.0; R v3.5.1; RStudio v1.1.463; Perl v5.10.1; TIDE v2.0.1,R v3.5.1; Microsoft Excel 16.16.2. Custom scripts have been deposited in https://github.com/yuwei4891/DerianoLab-HTGTS.git

For manuscripts utilizing custom algorithms or software that are central to the research but not yet described in published literature, software must be made available to editors and reviewers. We strongly encourage code deposition in a community repository (e.g. GitHub). See the Nature Research guidelines for submitting code & software for further information.

Data

Policy information about availability of data

All manuscripts must include a data availability statement. This statement should provide the following information, where applicable:

- Accession codes, unique identifiers, or web links for publicly available datasets
- A list of figures that have associated raw data
- A description of any restrictions on data availability

The LAM-HTGTS and Igk capture sequencing data have been deposited in NCBI's Gene Expression Omnibus under accession number GSE138137.
The source data underlying Figures 1B, 1C, 1E, 3B, 3D, 4A, 4B, 5A, 5B, 5C, 5D, 5F and Supplementary Figures S1A, S1B, S1C, S1D, S2D, S4A, S6B, S7C, S8B, S8C, S8D, S8E, S9B, S10A, S10B, S11, S12A, S12B, S12C, S13A, S13B, S13C, S14A, S14B, S15B are provided as a Source Data file. Coding and signal sequences of mouse Igk V and Igk J segments were downloaded from MGI database (http://www.informatics.jax.org/). Repetitive genomic coordinate for mouse reference genome were downloaded from NCBI Table Brower (http://genome.ucsc.edu/cgi-bin/hgTables)

Field-specific reporting

Please select the one below that is the best fit for your research. If you are not sure, read the appropriate sections before making your selection.

☒ Life sciences ☐ Behavioural & social sciences ☐ Ecological, evolutionary & environmental sciences

For a reference copy of the document with all sections, see nature.com/documents/nr-reporting-summary-flat.pdf

Life sciences study design

All studies must disclose on these points even when the disclosure is negative.

Sample size	Sample sizes were determined using power analysis to determine minimum sample sizes required.
Data exclusions	No data was excluded from this study.
Replication	All experiments were repeated by at least two biological replicates with two or three isogenetic clones with consistent results.
Randomization	Randomization of samples was taken during animal experiments, mice were selected for treatment group based on a unique mouse ID which does not indicate specific information. Age-matched mice were used as a method to control.
Blinding	Analysis of mice experiment and metaphase FISH was blinded to prevent bias.

Reporting for specific materials, systems and methods

We require information from authors about some types of materials, experimental systems and methods used in many studies. Here, indicate whether each material, system or method listed is relevant to your study. If you are not sure if a list item applies to your research, read the appropriate section before selecting a response.

Materials & experimental systems

n/a	Involved in the study	
☐	☒	Antibodies
☐	☒	Eukaryotic cell lines
☒	☐	Palaeontology and archaeology
☐	☒	Animals and other organisms
☒	☐	Human research participants
☒	☐	Clinical data
☒	☐	Dual use research of concern

Methods

n/a	Involved in the study	
☒	☐	ChIP-seq
☐	☒	Flow cytometry
☒	☐	MRI-based neuroimaging

Antibodies

Antibodies used

Primary antibodies used in Western blot studies:
anti-P53: Santa Cruz sc-393031, clone A-1, 1:2000
anti-XRCC4: Santa Cruz sc-8285, clone C20, 1:1300
anti-γ-Tubulin: Sigma Aldrich T6557, clone GTU-88, 1:10000
anti-PARP1: Tissue culture supernatants of the mouse hybridoma clone A6.4.12 (Aoufouchi S. Monoclonal antibodies A4.3.4; A6.4.12; B5.3.9; B15.4.13 anti-poly(ADP-ribose)polymerase. Hybridoma. 1997; 16: 583), 1:50.
anti-β-Actin: Sigma Aldrich A1978, clone AC-15, 1:5000
anti-Vinculin: Santa Cruz sc-73614, clone 7F9, 1:1000
anti-GFP: Santa Cruz sc-9996, clone B-2, 1:1000

Secondary anti-IgG antibodies used in Western blot studies:
IRDye® 800CW Goat anti-Mouse IgG: LI-COR 926-32210, 1:10000
IRDye® 680RD Goat anti-Mouse IgG: LI-COR 926-68070, 1:10000 for Vinculin, 1:20000 for γ-Tubulin
IRDye® 800CW Donkey anti-Goat IgG: LI-COR 926-32214, 1:10000
HRP-linked anti-Mouse IgG: Cell Signaling Technology 7076, 1:10000

Antibodies used in Flow Cytometry studies:
anti-hCD4-PE: Miltenyi 130-113-254, clone M-T466, 1:100
anti-CD19: BD Biosciences 560375, clone 1D3, 1:200
anti-CD43: BD Biosciences 553271, clone S7, 1:200
anti-Th1.1: BD Biosciences 561409, clone OX-7, 1:200

Validation

Validation:
anti-P53: validated by Santa Cruz and suitable for western blot assays against mouse protein.

anti-XRCC4: validated by Santa Cruz and suitable for western blot assays against mouse protein.
anti-γ-Tubulin: validated by Sigma and suitable for western blot assays against mouse protein.
anti-PARP1: Aoufouchi S. Monoclonal antibodies A4.3.4; A6.4.12; B5.3.9; B15.4.13 anti-poly(ADP-ribose)polymerase. Hybridoma. 1997; 16: 583.
anti-β-Actin: validated by Sigma and suitable for western blot assays against mouse protein.
anti-Vinculin: validated by Santa Cruz and suitable for western blot assays against mouse protein.
anti-GFP: validated by Santa Cruz and suitable for western blot assays against mouse protein.
All antibodies used in flow cytometry studies were validated by the manufacturers as suitable for use in flow cytometry assays against specific antigens/markers.
anti-hCD4-PE: validated by Miltenyi and suitable for flow cytometry experiments against human, non-human primate protein.
anti-CD19: validated by BD Biosciences and suitable for flow cytometry experiments against mouse protein.
anti-CD43: validated by BD Biosciences and suitable for flow cytometry experiments against mouse protein.
anti-Th1.1: validated by BD Biosciences and suitable for flow cytometry experiments against mouse protein.

Eukaryotic cell lines

Policy information about cell lines

Cell line source(s)

Murine derived v-Abl/Bcl2 pro-B cell lines were generated as previously described (Lenden Hasse, H. et al. Generation and CRISPR/Cas9 editing of transformed progenitor B cells as a pseudo-physiological system to study DNA repair gene function in V(D)J recombination. J Immunol Methods 451, 71-77 (2017); Lescale, C. et al. RAG2 and XLF/Cernunnos interplay reveals a novel role for the RAG complex in DNA repair. Nat Commun 7, 10529 (2016)). Briefly, total bone marrow from 3–5-week-old mice was cultured and infected with a retrovirus encoding v-Abl kinase to generate immortalized pro-B cell lines. v-abl transformed pro-B cell lines were then transduced with pMSCV-Bcl2-puro retrovirus to protect them from v-abl kinase inhibitor-induced cell death.

CRISPR-Cas9 edited v-Abl/Bcl2 pro-B cell clones were generated as previously described (Lenden Hasse, H. et al. Generation and CRISPR/Cas9 editing of transformed progenitor B cells as a pseudo-physiological system to study DNA repair gene function in V(D)J recombination. J Immunol Methods 451, 71-77 (2017); Lescale, C. et al. Specific Roles of XRCC4 Paralogs PAXX and XLF during V(D)J Recombination. Cell Rep 16, 2967-2979 (2016)).

List of cell lines used in this study:
12095: WT, murine-derived pro-B cells (Lescale C. et al., Nat Commun. 2016)
12096: WT, murine-derived pro-B cells (Lescale C. et al., Nat Commun. 2016)
6920.1: p53-/-, murine-derived pro-B cells (Lescale C. et al., Nat Commun. 2016)
6943.1: p53-/-, murine-derived pro-B cells (Lescale C. et al., Nat Commun. 2016)
15307: p53-/-, murine-derived pro-B cells (Lescale C. et al., Nat Commun. 2016)
Xr95-22: Xrcc4-/-, CRISPR-Cas9 knockout pro-B cell clone (Lescale C., et al. Cell Reports. 2016)
Xr95-23: Xrcc4-/-, CRISPR-Cas9 knockout pro-B cell clone (Lescale C., et al. Cell Reports. 2016)
Xr95-50: Xrcc4-/-, CRISPR-Cas9 knockout pro-B cell clone (Lescale C., et al. Cell Reports. 2016)
Xr15307-3: Xrcc4-/- p53-/-, CRISPR-Cas9 knockout pro-B cell clone (This study)
Xr15307-11: Xrcc4-/- p53-/-, CRISPR-Cas9 knockout pro-B cell clone (This study)
PolQ-2-307: Polq-/- p53-/-, CRISPR-Cas9 knockout pro-B cell clone (This study)
PolQ-5-307: Polq-/- p53-/-, CRISPR-Cas9 knockout pro-B cell clone (This study)
PolQ-2-307-Xr25: Xrcc4-/- Polq-/- p53-/-, CRISPR-Cas9 knockout pro-B cell clone (This study)
PolQ-2-307-Xr211: Xrcc4-/- Polq-/- p53-/-, CRISPR-Cas9 knockout pro-B cell clone (This study)
Xr307-3-PolQ35: Xrcc4-/- Polq-/- p53-/-, CRISPR-Cas9 knockout pro-B cell clone (This study)
17585: Rag2-/- p53-/-, murine-derived proB cells (This study)
17587: Rag2-/- p53-/-, murine-derived proB cells (This study)
Xr-17585-17: Rag2-/- Xrcc4-/- p53-/-, CRISPR-Cas9 knockout pro-B cell clone (This study)
Xr-17585-18: Rag2-/- Xrcc4-/- p53-/-, CRISPR-Cas9 knockout pro-B cell clone (This study)
PolQ-17587-8: Rag2-/- Polq-/- p53-/-, CRISPR-Cas9 knockout pro-B cell clone (This study)
PolQ-17587-13: Rag2-/- Polq-/- p53-/-, CRISPR-Cas9 knockout pro-B cell clone (This study)
PolQ-17587-22: Rag2-/- Polq-/- p53-/-, CRISPR-Cas9 knockout pro-B cell clone (This study)
X-P-87-4-4-X-3: Rag2-/- Xrcc4-/- Polq-/- p53-/-, CRISPR-Cas9 knockout pro-B cell clone (This study)
X-P-87-4-4-X-14: Rag2-/- Xrcc4-/- Polq-/- p53-/-, CRISPR-Cas9 knockout pro-B cell clone (This study)
6284: Parp1-/- p53-/-, murine-derived pro-B cells (Galindo-Campos M.A., et al. Cell Death & Differentiation. 2019)
6286: Parp1-/- p53-/-, murine-derived pro-B cells (Galindo-Campos M.A., et al. Cell Death & Differentiation. 2019)
Xr-P1P2Fp53-2: Parp1-/- Xrcc4-/- p53-/-, CRISPR-Cas9 knockout pro-B cell clone (This study)
Xr-P1P2Fp53-3: Parp1-/- Xrcc4-/- p53-/-, CRISPR-Cas9 knockout pro-B cell clone (This study)
Xr-P1P2Fp53-4: Parp1-/- Xrcc4-/- p53-/-, CRISPR-Cas9 knockout pro-B cell clone (This study)

Authentication

Parp1, Xrcc4 and p53 gene knock out in CRISPR-Cas9 knockout pro-B cell clones and murine-derived pro-B cells were validated by PCR, sanger sequencing and western blotting.
Polq gene knock out in CRISPR-Cas9 knockout pro-B cell clone was validated by PCR and sanger sequencing, as there is no Polq antibody available.
WT pro-B cell clones (12095, 12096) were generated from the bone marrow of WT mouse and their genotype was validated by PCR and western blotting.

Mycoplasma contamination

The cell lines were not tested for mycoplasma contamination.

Commonly misidentified lines
(See ICLAC register)

No commonly misidentified cell lines were used in this study.

Animals and other organisms

Policy information about studies involving animals; ARRIVE guidelines recommended for reporting animal research

Laboratory animals	Rag2-/- mice (Taconic, strain 129S/SvEv-Gpi1c) were bred with p53+/- mice (Jackson laboratory, strain B6.129S2) to generate doubly deficient mice used to produce v-Abl transformed Rag2-/- p53-/- pro-B cell lines (Bianchi, J.J., Murigneux, V., Bedora-Faure, M., Lescale, C. & Deriano, L. Breakage- Fusion-Bridge Events Trigger Complex Genome Rearrangements and Amplifications in Developmentally Arrested T Cell Lymphomas. Cell Rep 27, 2847-2858 e2844 (2019)). 8-weeks-old males Rag2-/- p53-/- mice were used to produce v-Abl transformed Rag2-/- p53-/- pro-B cell lines #17585 and #17587.<br>6-8 weeks-old males and females Rag2-/- mice (mixed strain B6.129S2, 129S/SvEv-Gpi1c) were used for tumor cell injections.
Wild animals	The study did not involve wild animals.
Field-collected samples	The study did not involve samples collected from the field.
Ethics oversight	All experiments were performed in accordance with the guidelines of the institutional animal care and ethical committee of Institut Pasteur/CETEA n°89 under the protocol numbers 180006/14778 and 170027.

Note that full information on the approval of the study protocol must also be provided in the manuscript.

Flow Cytometry

Plots

Confirm that:

☒ The axis labels state the marker and fluorochrome used (e.g. CD4-FITC).

☒ The axis scales are clearly visible. Include numbers along axes only for bottom left plot of group (a 'group' is an analysis of identical markers).

☒ All plots are contour plots with outliers or pseudocolor plots.

☒ A numerical value for number of cells or percentage (with statistics) is provided.

Methodology

Sample preparation	For V(D)J recombination assays, purified pMX-INV v-Abl pro-B cells were treated with 3 µM of the Abl kinase inhibitor STI-571 for 72 h, washed with PBS +2% FBS and stained with hCD4 antibody at 4°C for 30 min.<br><br>For cell cycle assays, cells were incubated with 100 µM Edu for 30 min at 37°C and washed once with PBS. Cells were then fix cells with 4% Paraformaldehyde for 30 min at room temperature and washed once with PBS. Cells were stained with 1mM CuSO4, 2 µM iFluor™ 647 azide and 100mM L-Ascorbic acid for 1 hour at room temperature. After one wash with PBS, cells were incubated with PI staining cocktail (1.9mM sodium citrate tribasic dehydrate, 25 µg/ml propidium iodide, 250 µg/ml RNAse A, 0.5mM Tris-Hcl, 0.75mM NaCl) for 1 hour at 37°C. Finally, cells were centrifuged and resuspended in PBS for FACS analysis.<br><br>For mouse analysis, single-cell suspensions of spleen, bone marrow, lymph node, and thymus were washed with PBS +2% FBS and stained with cell surface markers at 4°C for 30 min. Cells were then washed and resuspended in PBS +2% FBS before acquisition.
Instrument	Samples were acquired on a BD FACS Canto II and LSR Fortessa (BD Biosciences).
Software	Samples were analysed using Flowjo v10 .
Cell population abundance	After transfection of Cas9-GFP and guide RNA plasmids, GFP positive cells were sorted into single cell for the selection of knock out clones.
Gating strategy	For V(D)J recombination assays, live cells were gated using FSC-A/SSC-A, then single cells were gated using FSC-H/FSC-W. V(D)J recombination levels were scored as the percentage of GFP positive cells among total hCD4 positive cells.<br><br>For cell cycle assays, fixed single cells were gated using FSC-A/SSC-A and then FSC-H/FSC-W. G1 cells were defined as Edu- PI-, S-phase cells were defined as Edu+ and G2/M cells were defined as EdU- PI+.<br><br>For mouse analysis, live lymphocytes cells were gated using FSC-A/SSC-A, then single cells were gated using FSC-H/FSC-W. Engrafted v-Abl pro-B cells were identified as CD19+ CD43+.

☒ Tick this box to confirm that a figure exemplifying the gating strategy is provided in the Supplementary Information.
